# Distribution and Levels of Insulin-like Growth Factor 2 Receptor Across Mouse Brain Cell Types

**DOI:** 10.3390/receptors5010001

**Published:** 2025-12-23

**Authors:** Jessica R. Gaunt, Gokul Manoj, Cristina M. Alberini

**Affiliations:** Center for Neural Science, New York University, New York, NY 10003, USA;

**Keywords:** insulin-like growth factor 2 receptor, cation-independent mannose 6 phosphate receptor, brain, mouse, cell types, hippocampus

## Abstract

**Background:**

The insulin-like growth factor 2 receptor (IGF-2R), also known as the cation-independent mannose 6-phosphate receptor (CI-M6PR), is emerging as a critical receptor for brain function and disease. IGF-2R, in fact, plays a key role in long-term memory, and its activation by several ligands shows beneficial effects in multiple neurodevelopmental and neurodegenerative disease models. Thus, its targeting is very promising for neuropsychiatric therapeutic interventions. IGF-2R’s main known functions are transport of lysosomal enzymes and regulation of developmental tissue growth, but in the brain, it also controls learning-dependent protein synthesis underlying long-term memory. However, little is known about this receptor in brain cells, including its cell-type-specific and subcellular expression.

**Methods:**

We conducted a comprehensive investigation to comparatively assess IGF-2R protein levels in different brain cell types across various brain regions in adult male C57BL/6J mice using dual and multiplex immunofluorescent staining with cell-type-specific markers. The IGF-2R protein distribution was also compared with *Igf2r* mRNA expression in publicly available single-cell RNA sequencing databases.

**Results:**

A ranking of IGF-2R levels in the soma of various cell types in the hippocampus and cortical regions revealed that the highest enrichment is, by far, in excitatory and inhibitory neurons, followed by vascular mural cells and subpopulations of oligodendrocyte lineage cells, with low to undetectable levels in astrocytes, microglia, vascular endothelial cells, and perivascular fibroblasts. High levels of IGF-2R were also found in ependymal cells, choroid plexus epithelial cells, and a subpopulation of meningeal fibroblast-like cells. IGF-2R was found in dendritic and putative axonal compartments throughout the brain, with particularly high levels in the stratum lucidum. The receptor’s protein distribution aligned with that of the mRNA in mouse brain databases.

**Conclusions:**

These results suggest that IGF-2R-mediated functions in the brain vary across different cell types and subcellular compartments, with the most active roles in specific subpopulations of neurons, mural cells, ependymal cells, meningeal cells, and cells of the oligodendrocyte lineage. This study advances our understanding of IGF-2R’s distribution in the brain, which is essential for formulating new hypotheses about its functions and therapeutic targeting.

## 1. Introduction

Insulin-like growth factor 2 receptor (IGF-2R), also known as cation-independent mannose-6-phosphate receptor (CI-M6PR), is a single-transmembrane protein receptor of approximately 300 kDa that lacks a tyrosine kinase domain and has two major ligands: IGF-2 and M6P-conjugated proteins [1,2]. Global *Igf2r* knockout in mice increases IGF-2 levels, causing larger body size, organ enlargement, cardiac issues, and death within 2 weeks of birth, indicating the receptor’s role in limiting tissue growth [3,4]. In adult tissues, the contributions of IGF-2R have been poorly studied and mostly considered in the context of its intracellular distribution, which is within the secretory pathway across the Golgi apparatus, trans-Golgi network (TGN), endosomes, and plasma membrane [5,6]. One primary function of IGF-2R is to bind newly synthesized M6P-conjugated lysosomal enzymes and target them to lysosomes [7]. Because of this essential cellular function, IGF-2R is believed to be expressed by all cell types; however, experimental evidence of such expression and comparisons of IGF-2R levels across different cell types are lacking. A smaller 46 kDa cation-dependent M6P receptor (CD-M6PR) exists and also binds M6P-conjugated lysosomal hydrolases in the TGN and delivers them to pre-lysosomal compartments [8]. Thus, the two M6PRs may be redundant or differentially regulated in various tissues and cell types. Notably, both receptors are found at relatively high levels in the brain, where their functions and cell-type-specific expression have yet to be characterized.

IGF-2R in hippocampal neurons plays a crucial role in long-term memory formation, as evidenced by the loss of long-term memory following its in vivo pharmacological blockade or knockout [2]. Conversely, IGF-2R activation via intracerebral or systemic administration of IGF-2 or M6P significantly enhances memory retention and persistence [2,9–13]. These ligands, through IGF-2R, also reverse memory and other behavioral impairments in aged rodents and in various rodent models of neurodevelopmental disorders and neurodegenerative diseases (reviewed in [14]), indicating that the receptor governs biological pathways essential for healthy brain function. Notably, neuropsychiatric diseases that respond positively to IGF-2R agonists share a common problem: the overaccumulation of proteins, along with alterations in protein degradation pathways, such as those mediated by autophagy or endosomal acidification [15]. This dysregulated protein metabolism eventually leads to impaired plasticity, which can result in neurodegeneration [16–19]. Thus, IGF-2R in the brain, through its role in controlling lysosomal functions and protein metabolism homeostasis, has emerged as a potential key target for promoting memory enhancement and preventing or treating aging-related cognitive impairments, neurodevelopmental disorders, and neurodegenerative diseases [14]. In line with these findings, IGF-2 via IGF-2R promotes neuronal survival and protects against neural insults [20–22].

Despite these vital functions in the brain, and the fact that in both human and rodent brains IGF-2R has been primarily found in neurons of the cortex, hippocampus, and thalamus, as well as in the choroid plexus [2,6,23–27], our understanding of IGF-2R’s brain-wide distribution remains incomplete, and its expression in different cell types is especially unclear. Thus, in this study, we first assessed the distribution of IGF-2R across adult mouse brain regions and compared it to CD-M6PR. Then, focusing on areas critical for learning and memory, i.e., dorsal hippocampus (dHC), and medial prefrontal (mPFC), anterior cingulate (ACC), and retrosplenial (RSC) cortices, we analyzed IGF-2R levels in several cell populations identified by cell-type-specific markers, employing confocal imaging of dual-stained tissues and relative quantification of multiplex immunofluorescence. Specifically, we assessed IGF-2R levels in excitatory, inhibitory, and cholinergic neurons, oligodendrocytes/oligodendrocyte precursor cells (OPCs), microglia, astrocytes, vascular endothelial cells, mural cells, and fibroblasts. Analyses of cell populations in the ventricles and meninges were also conducted. Finally, we compared our findings to the distribution of *Igf2r* mRNA across mouse brain cell populations in public single-cell RNA-Seq databases.

## 2. Materials and Methods

### 2.1. Animals

Adult male C57BL/6J mice (postnatal day [PN] 80–120) were used for all experiments. Mice were group-housed in individually ventilated cages with nesting materials and paper huts on a 12:12 h light-dark cycle. Subjects were handled for 5 days for 3.5 min before any procedure. In total, twelve animals were used in this study. All protocols complied with the National Institutes of Health Guide for the Care and Use of Laboratory Animals and were approved by the New York University Animal Welfare Committee.

### 2.2. Immunohistochemistry and Confocal Microscopy Analyses

Briefly, mice (N = 6) were anesthetized with an intraperitoneal injection of chloral hydrate (750 mg/kg) and transcardially perfused with 1× phosphate-buffered saline (PBS) followed by 4% paraformaldehyde (PFA) in 1× phosphate buffer (0.1 M PB). The brains were post-fixed in 4% PFA overnight at 4 °C, followed by 30% sucrose in 1× PBS (pH 7.4) for 48–72 h. 20 μm cryosections in the sagittal (N = 3) or coronal (N = 3) planes were collected for free-floating immunofluorescent staining and stored at −20 °C in cryoprotectant solution [50% 0.1 M PB, pH 7.4; 25% ethylene glycol, 25% glycerol].

Rabbit recombinant monoclonal antibodies were used to target IGF-2R (1:16,000, EPR6599, ab124767, Abcam, Cambridge, UK) and CD-M6PR (1:1000, EPR7691, ab134153, Abcam, Cambridge, UK). The specificities of both antibodies were confirmed by knockout in HAP1 cells by the manufacturer. Additional specificity tests were conducted in the mouse brain using a viral-mediated knockout of IGF-2R [2]. Primary antibodies used to identify cell types and compartments are listed in Table 1. Antigen retrieval, performed by incubation at 100 °C for 5 min in 0.01M citrate buffer [pH 6, 0.05% Tween 20], was necessary to observe specific staining with the antibody to ALDH1L1. Sections were then incubated in blocking solution [1× PBS, pH 7.4 with 0.25% Triton X-100, 5% normal goat or donkey serum, 1% bovine serum albumin] for 2 h at room temperature, followed by incubation with the primary antibodies diluted in blocking solution at 4 °C overnight. Following primary antibody incubations, sections were washed 3 times for 10 min in 1× PBST [1× PBS, pH 7.4 with 0.25% Triton X-100], and then incubated with secondary antibodies in PBST for 2 h at room temperature. The following Alexa Fluor conjugated secondary antibodies (Thermo Fisher Scientific, Waltham, MA, USA) were used at a dilution of 1:1000: goat anti-rabbit 488 highly cross-adsorbed plus (A32731), goat anti-mouse 488 highly cross-adsorbed plus (A32723), goat anti-rabbit 488 (A11034), goat anti-chicken 488 (A11039), donkey anti-rat 488 (A21208), goat anti-guinea pig 488 (A11073), goat anti-rabbit 568 (A11036), donkey anti-goat 488 (A11055), and donkey anti-rabbit 647 (A31573). The sections were washed 3 times for 10 min in PBST, then incubated with 4′,6-diamidino-2-phenylindole (DAPI, 300 μM stock, 1:1000) in PBS for 5 min and washed in PBS for 10 min. Sections were mounted with Prolong Diamond antifade mounting medium (P36961, Invitrogen, Waltham, MA, USA). Two sections per brain region for each combination of antibodies were analyzed.

Widefield images of whole sections were captured at 10× magnification using an Olympus VS120 virtual slide scanner microscope (Olympus, Center Valley, PA, USA). Confocal single-plane or z-stack images were captured at 20× or 63× magnification using a Leica SP8 confocal microscope (Leica, Wetzlar, Germany) at 1024 × 1024 pixel resolution with a line average of 2. Images were processed and quantified using the Fiji software v2.16.0 (National Institutes of Health, Bethesda, MD, USA) [28]. All confocal images reported in this manuscript show a representative single z-plane, and image intensity was adjusted for display.

To quantify IGF-2R and CD-M6PR immunostaining across regions in sagittal sections, immunofluorescence intensity was measured in arbitrary units (AU), which were defined by the raw grayscale pixel values detected by the camera under fixed imaging conditions. Regions of interest (ROIs) for relative abundance quantifications across brain regions were identified according to the Allen Brain Atlas [29]. The mean intensity in each ROI was normalized by subtracting the background intensity measured in the corresponding ROI of control sections, which were incubated in parallel without the primary antibody and imaged using the same parameters. The same secondary antibody and imaging conditions, and therefore negative control images, were used for both primary antibodies. To account for cell density, the total intensity in each ROI (i.e., normalized mean intensity multiplied by area) was divided by the number of DAPI+ nuclei. To count nuclei, a difference of Gaussians filter was applied to images of DAPI staining, then binary images were generated by applying an intensity threshold determined by the triangle algorithm; overlapping nuclei were separated using the watershed algorithm, and particles within a size range of 10 to 250 pixels were counted. The accuracy of DAPI counts based on nuclear masks was ensured by visual inspection, and ROIs with unreliable quantification due to high cell density were excluded.

### 2.3. Colocalization Analysis

Immunostained sections were prepared as described above. High-resolution images for quantification of IGF-2R in dendrites and spines were captured at 2048 × 2048 pixels in a single z-plane, averaging 16 images for MAP2 and 8 for PSD95. Laser power in the IGF-2R channel (568 nm) was set to capture relatively low intensity signals in processes (such that neuron somas were overexposed). The same parameters were used for all images in an experiment.

Quantification of colocalization was performed in Fiji using the BIOP JACoP plugin (https://github.com/BIOP/ijp-jacop-b; accessed on 28 March 2025) [30]. First, a background value for each marker was established by measuring mean intensities in areas with low expression of the selected markers, and this value was subtracted from all images. Neuron somas, which were identified by MAP2 and/or intense IGF-2R staining surrounding a DAPI+ nucleus, were excluded from ROIs for dendrites/spines. In images of the cortex, all DAPI+ nuclei were also excluded from quantification ROIs. The stratum oriens (SO), stratum radiatum (SR), and stratum moleculare (SM) were further divided into proximal, medial, and distal ROIs, approximately 0–30 μm, 30–60 μm and >70 μm from the stratum pyramidale (SP)_in CA1 and CA3 or stratum granulosum (SG) in the dentate gyrus (DG), respectively. Mander’s overlap coefficients for each ROI were calculated in background-subtracted images. Overlays of binary images were generated to display the overlap between channels for pixels with an intensity greater than zero, following background subtraction.

### 2.4. Multiplex Tyramide Signal Amplification Immunostaining

Following transcardial perfusion as described above, brains (N = 6) were post-fixed in 4% PFA overnight at 4 °C, then transferred to 70% ethanol. Tissues were dehydrated through a series of graded ethanol solutions, followed by xylene and then infiltrated with paraffin (Paraplast X-tra, 39603002, Leica, Wetzlar, Germany) on a Leica Peloris II tissue processor. Embedding was performed on a Leica Arcadia embedder. Coronal 5 μm sections were obtained on a microtome (RM2255, Leica, Wetzlar, Germany) at Bregma levels +2.2 to +1.4 mm (mPFC), +1.1 to 0.0 mm (ACC) and −1.5 to −2.2 mm (dHC and RSC) and mounted directly. For each panel, four slides containing sections from all three co-ordinates and two slides containing only dHC/RSC sections were stained (one subject/slide). Sections were immunostained on a BondRx autostainer (Leica, Wetzlar, Germany), according to the manufacturer’s instructions. In brief, sections were first deparaffinized and rehydrated, followed by an antigen retrieval step with either Bond epitope retrieval solution ER1 (AR9961, Leica, Wetzlar, Germany) or ER2 (AR9640) at 100 °C for either 20 or 60 min as indicated in Table 2. Peroxide treatment to inhibit endogenous peroxidases was then performed, followed by blocking. Slides were incubated with the first primary antibody for a duration of 30 min, except for antibodies to COL1A1, PDGFRβ and OLIG2, which were incubated for 60 min. Incubation with the Opal polymer horseradish peroxidase (HRP) secondary antibody (Akoya Biosciences, Marlborough, MA, USA) was then performed, followed by HRP-mediated tyramide signal amplification (TSA) with a specific Opal fluorophore for 10 min, except for Opal780, which was incubated for 60 min. A longer incubation time and higher concentration of primary antibody were used with Opal780 to compensate for the relatively weak signal of this fluorophore (Table 2). The primary and secondary antibodies were subsequently removed by heat-induced retrieval, leaving the Opal fluorophore covalently bonded to tyrosine residues surrounding the target epitope. These steps were repeated with subsequent primary and secondary antibody pairs with different Opal fluorophores (Table 2). Sections were counterstained with spectral DAPI (FP1490, Akoya Biosciences, Marlborough, MA, USA) and mounted with ProLong Gold Antifade (P36935, Thermo Fisher Scientific, Waltham, MA, USA). Antibody dilution and antigen retrieval conditions were optimized for each target prior to multiplex experiments in diaminobenzidine (DAB) and fluorescent single stainings. Semi-automated acquisition of widefield images was performed at 20× magnification on an Akoya PhenoImagerHT (formerly Vectra Polaris) multispectral imaging system, using PhenoImagerHT 2.0 software in conjunction with Phenochart 2.0 and InForm 3.0 (Akoya Biosciences, Marlborough, MA, USA) to generate unmixed whole-slide qptiff scans. Spectral unmixing to remove fluorescent crosstalk and autofluorescence was optimized using sections stained for each antibody with the corresponding fluorophore and DAPI, as well as unstained controls.

### 2.5. Quantification Using Machine Learning Algorithms

Analysis and quantification of multiplex images was performed using QuPath software v0.5.1 [31], employing machine learning algorithms to identify and classify cells. First, ROIs in the dHC, mPFC, ACC, RSC, and corpus callosum (CC; including the alveus) were manually annotated using the brush and wand tools, excluding areas with staining artefacts or tissue folds. Sections and/or areas with uneven IGF-2R staining or weak DAPI staining were also excluded; data from one subject was excluded for the mPFC, ACC and RSC in both panels, and dHC in panel 2. Nuclei were then identified in a two-step process. First areas of DAPI+ staining were classified using an artificial neural network (ANN) trained on manually annotated images sampled from all slides and ROIs [training parameters: resolution very high (0.99 μm/pixel); features Gaussian, Laplacian of Gaussian, and gradient magnitude; scales 0.5–4 μm; local normalization false; classification parameters: minimum object size 10 μm^2^, minimum hole size 20 μm^2^]. Then the positive cell detection tool was applied to DAPI+ areas to identify individual nuclei and expand each nucleus by 2 μm [parameters: pixel resolution 0.5 μm, background radius 8 μm, opening by reconstruction true, median filter radius 1.5 μm, Gaussian filter sigma 1.6 μm, minimum area 10 μm^2^, maximum area 100 μm^2^, intensity threshold 1.0, split by shape true, and smooth boundaries true]. Mean IGF-2R intensity was then measured in the expanded nuclei, which we refer to as discs. This approach was chosen to ensure a comparable method for all cell types, with the goal of capturing the area that in the various cell types is more intensely stained with IGF-2R and includes the Golgi apparatus. The cell detection method was subsequently modified for IBA1+ cells (see below). In total, N = 144,734 discs were included in the final analysis (Panel 1: dHC—26739, mPFC—11509, ACC—6736, RSC—7932, CC—9844; Panel 2: dHC—33253, mPFC—8804, ACC—5683, RSC—17619, CC—16615).

To classify discs by expression of cell type markers, pixel classifiers were trained to detect areas of positive staining for each marker and applied independently to classify disc centroids as positive or negative. Identification accuracy was confirmed for each image by visual inspection. Pixel classifiers were used—instead of object classifiers—to automate and standardize detection across images with variable staining intensity, while accounting for differences in the distributions and expression levels of markers across brain regions. Parameters were optimized—dependent on the staining distribution of each marker—to include nuclei surrounded by highly stained cytoplasm in positive classifications, while excluding relatively low intensity staining in cellular processes. The following default parameters were used and modified as necessary to improve classification accuracy: ANN algorithm, 0.99 μm/pixel resolution, Gaussian and Laplacian of Gaussian image transformations, and smoothing scale 1 μm or 0.5–8 μm. Local normalization was used with a scale of 32 μm for CaMK2α, GAD67, and PDGFRβ, and a scale of 20 μm for ALDH1L1. For CaMK2α, an additional classifier was used to increase the number of positive cells in the hippocampus, due to the dense packing of neuron somas compared to the cortex.

In cortical regions, IBA1+ and OLIG2+ discs frequently overlapped with neuron somas, and IGF-2R intensity was consequently spuriously high in these populations (Figure S1). As IBA1 staining with TSA was intense in somas and nuclei and substantially lower in processes, we were able to more accurately capture the shape and size of IBA1+ somas by using IBA1 staining itself instead of DAPI to detect these cells and thus reduce overlap with neurons. IBA1 staining in cell bodies was identified using a pixel classifier [training parameters: resolution very high; features Gaussian and Hessian determinant; scales 0.5–8 μm; local normalization false] and the positive cell detection tool was applied to IBA1+ areas. The same classification and detection parameters were used as for DAPI, except that the detection area was not expanded. In regions with little overlap between IBA1+ discs and neuron somas, i.e., the dHC and CC, mean IGF-2R intensities were not significantly different between methods (*t*-tests; Figure S1), and in all regions, mean IGF-2R intensity in IBA1+ somas detected using the latter method was similar to that of ALDH1L1+ discs, in line with our observations in confocal double staining experiments, thus supporting the validity of using this method for comparison with other cell types. This approach also increased the number of IBA1+ cells detected. The overlap between cortical neurons and OLIG2+ somas could not be resolved due to both the nuclear localization of OLIG2 and the greater degree of overlap between these populations [32].

To subtract diffuse IGF-2R staining in neuronal processes, we used an ANN to divide each ROI into areas of ‘high IGF-2R’ containing IGF-2R+ cell bodies, and ‘low IGF-2R’, containing diffuse or absent staining [training parameters: resolution very high; features Gaussian, Laplacian of Gaussian, and weighted standard deviation; scales 0.5–8 μm; local normalization scale 32 μm; classification parameters: minimum object size 0 μm^2^, minimum hole size 0 μm^2^]. Within each ROI, the median intensity of the ‘low IGF-2R’ area was then measured and subtracted from the mean IGF-2R intensities of discs.

For summary statistics, discs positive for only one marker were identified. We excluded discs positive for glial and vascular markers in the dHC SP and SG to prevent overlap with neuron somas, and excluded CaMK2α+ discs in the CC and subregions of the dHC that contain few to no excitatory neuron somas. Mean IGF-2R intensities of discs were normalized by dividing by the upper quartile of IGF-2R intensity across all DAPI+ discs in the same ROI within each image.

### 2.6. Analyses of Public Single-Cell RNA-Seq Databases

Publicly available comprehensive single-cell sequencing datasets profiling the mouse brain were identified. The datasets were (1) single-cell RNA-Seq of the whole juvenile (PN12–30) mouse brain, using male and female CD-1 and Swiss outbred strains (34 subjects), as well as juvenile and adult Vgat-Cre:tdTomato transgenic mice (6 subjects), which identified 265 cell populations from >0.5 million cells (http://mousebrain.org/; accessed on 26 February 2025) [33]; (2) single-nucleus RNA-Seq of the whole brain of adult (PN56) male and female C57BL/6J mice (55 subjects), which identified 4998 cell populations from >4.3 million nuclei (accessible at http://www.braincelldata.org/; accessed on 27 February 2025) [34], and (3) single-cell RNA-Seq of 9 regions of adult (PN60–70) male C57BL/6N mouse brain (3–14 subjects per brain region), which identified 565 populations from 690,000 cells (accessible at http://dropviz.org/; accessed on 17 March 2025) [35]. Further details of these datasets are available in the Supplementary Information of the corresponding publications.

Aggregate data comprising the average gene expression values for all cell populations (clusters) identified in each study were downloaded from the respective websites. Cell populations derived from the spinal cord and peripheral organs were removed from the dataset of Zeisel et al. [33], and the DC, Granulocyte, Lymphocyte, Myeloid, Olfactory Ensheathing, and Pituitary populations were removed from the dataset of Langlieb et al. [34]. Cell populations were then grouped into major cell type classes: annotations by the respective authors were validated by confirming expression of canonical marker genes and clustering in UMAPs, and reassigned where necessary to ensure consistency between datasets. Non-neuronal populations were categorized as follows, with canonical markers in brackets: astrocytes (*Aldh1l1*), ependymal (including choroid plexus epithelial cells; *Ttr*, *Foxj1*, *Aqp1*), vascular endothelial (*Pecam1*), mural (*Pdgfrb*, *Acta2*), fibroblasts (*Dcn*, *Col1a1*, *Pdgfra*), OPCs (*Sox10*, *Pdgfra*, *Olig2*), oligodendrocyte (*Olig2*, *Sox10*, *Mog*), immune (i.e., microglia and macrophage; *Aif1*), and neuroblasts (*Sox4*). Neurons were divided into four subpopulations according to gene expression and expression of neurotransmitters as labelled by the respective authors as follows: excitatory (VGLUT1/*Slc17a7*, VGLUT2/*Slc17a6*), inhibitory (GABA, glycine, *Gad1*, *Gad2*), cholinergic (acetylcholine, *Chat*), and other neurons (monoamines, i.e., serotonin, dopamine, noradrenaline; mixed expression of excitatory and inhibitory neurotransmitters; peptidergic neurons, and Cajal Retzius cells). Mean and SEM of *Igf2r* and *M6pr* expression were calculated across subpopulations for each class using the published normalized expression values for each dataset. Differential expression testing for the Saunders dataset was performed using the web tool provided by the authors. Single-cell RNA-Seq data targeting brain vascular populations were downloaded from https://betsholtzlab.org/VascularSingleCells/database.html (accessed on 27 February 2025) [36]. This dataset was obtained from transgenic reporter mice of the following genotypes: Pdgfrb-GFP;Cspg4-DsRed (2 subjects), Cldn5-GFP;Cspg4-DsRed (2 subjects), Pdgfra-H2BGFP (3 subjects), and SM22-Cre;R26-stop-tdTomato (3 subjects) on a C57BL6/J background, using fluorescence-activated cell sorting to isolate vascular fragments from the whole brains of 10-to 19-week-old mice of both sexes.

### 2.7. Experimental Design and Statistical Analyses

Data were analyzed using R v4.4.1 [37]. A within-subjects design was used for all experiments, with between three and six independent biological replicates used for quantification of single and multiplex immunostaining experiments, as indicated in figure legends. The number of subjects used in our experiments was the minimum required to obtain statistical significance, based on our previous studies and those used in similar studies [38]. To analyze multiplex TSA data, one-way ANOVAs followed by Tukey’s post hoc tests were performed for each brain region to determine the effect of cell type marker on IGF-2R intensity, using mean normalized values for each replicate. Two-way ANOVAs with Tukey’s post hoc tests were also performed using marker and region as factors to compare (1) normalized IGF-2R intensity of glial and vascular populations across dHC subregions, and (2) raw IGF-2R intensity of glial populations in the CC and dHC, using subject as a blocking factor to account for technical variability between replicates. A significance threshold of *p* < 0.05 was used for all tests. Normality of residuals was assessed using Shapiro–Wilk tests, and homogeneity of variance was assessed using Levene’s test. Mauchly’s test of sphericity was used for the within-subjects analysis. Although some groups showed mild departures from normality (*p* < 0.05), residuals were approximately symmetric and homoscedastic. Given the robustness of ANOVA to moderate non-normality, parametric tests were used. Full results of the statistical tests are provided in Supplementary Tables S1–S3. All data and materials produced in this study are available from the corresponding author (C.M.A.) upon request.

## 3. Results

### 3.1. Distribution of IGF-2R Across Mouse Brain Regions

**Figure 1.**
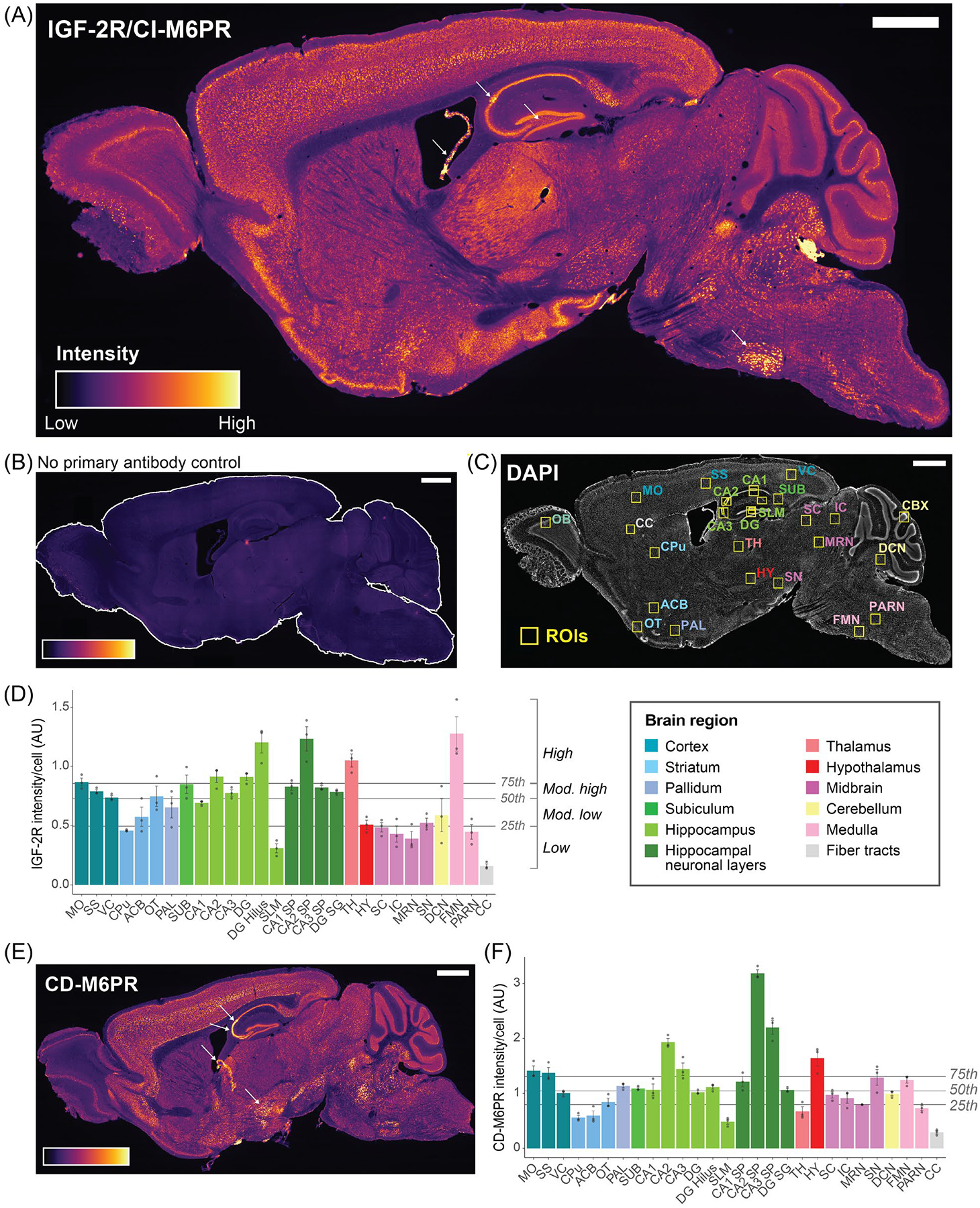
IGF-2R is detected throughout the mouse brain at differing levels across regions. (**A**) Representative 10× widefield image of IGF-2R immunofluorescent staining intensity in a sagittal section of adult mouse brain. Arrows indicate the ROIs with the highest staining intensities, including choroid plexus. All scale bars = 1 mm. (**B**) Representative image of background staining intensity in control sections, which lacked the primary antibody. (**C**) DAPI staining of the brain section in (**A**) for nuclei counting, annotated with ROIs. Annotations and color codes correspond to regions in the Allen Brain Atlas [29]. In the hippocampus (green labels), additional smaller ROIs (inner rectangles) indicate layers within subfields, i.e., the stratum pyramidale in CA1–3, and the stratum granulosum (top) and hilus (bottom) in the dentate gyrus (DG). (**D**) Mean IGF-2R staining intensity in arbitrary units (AU) divided by DAPI+ cell counts in each ROI (N = 3 anti-IGF-2R, N = 2 Control). Data are presented as mean ± SEM and points correspond to individual subject means. Olfactory bulb (OB) and cerebellar cortex (CBX) were excluded as high cell density precluded accurate quantification of cell number by DAPI+ staining. Quartiles classifying the distribution into low, moderately (mod.) low, moderately high, and high intensity levels are indicated (25th, 50th, and 75th percentiles). (**E**) Representative image of CD-M6PR immunostaining intensity. Arrows indicate the ROIs with the highest staining intensities, including choroid plexus. (**F**) Mean CD-M6PR intensity/cell in each ROI, with quartiles indicated as in (**D**) (N = 3 anti-CD-M6PR, N = 2 Control). OB—Olfactory Bulb; MO—Somatomotor Cortex; SS—Somatosensory Cortex; VC—Visual Cortex; CPu—Caudoputamen; ACB—Nucleus Accumbens; OT—Olfactory Tubercle; PAL—Pallidum; SUB—Subiculum; CA1–3—Cornu Ammonis 1–3; DG—Dentate Gyrus; SLM—Stratum Lacunosum Moleculare; SP—Stratum Pyramidale; SG—Stratum Granulosum; TH—Thalamus; HY—Hypothalamus; SC—Superior Colliculus; IC—Inferior Colliculus; MRN—Midbrain Reticular Nucleus; SN—Substantia Nigra; CBX—Cerebellar Cortex; DCN—Deep Cerebellar Nuclei; FMN—Facial Motor Nucleus; PARN—Parvicellular Reticular Nucleus; CC—Corpus Callosum.

Sagittal mouse brain sections were immunostained with an anti-IGF-2R recombinant monoclonal antibody (Figure 1A), the specificity of which had been confirmed by IGF-2R knockout [2]. Relative quantifications of IGF-2R immunofluorescence intensity were normalized by subtracting the background measured in secondary antibody-incubated (control) sections (Figure 1B) and expressed as mean intensity per cell, calculated by dividing the total ROI intensity by the number of nuclei identified with DAPI staining (Figure 1C).

IGF-2R was detected throughout the mouse brain, with varying levels in different regions, subregions, and layers (Figure 1). The IGF-2R staining intensity was divided into four levels according to quartiles, i.e., high (75–100th), moderately high (50–75th), moderately low (25–50th), and low (0–25th), with the 0, 25, 50, 75 and 100th percentiles, respectively, corresponding to fluorescence intensity/cell values of 0.141, 0.495, 0.730, 0.856, and 1.569 arbitrary units (AU) (Figure 1D; N = 3). High IGF-2R levels were found in the CA2 SP and hilus of the hippocampus, facial motor nucleus in the medulla, thalamus, and motor cortex (Figure 1D). A moderately high level of IGF-2R intensity/cell was found in the somatosensory and visual cortices, hippocampus CA1 and CA3 SP and DG SG, subiculum, and olfactory tubercle. We were unable to obtain mean intensity/cell values in the olfactory bulb and cerebellar cortex due to the high cell density in the granule layers, which precluded accurate counting using DAPI staining. However, both regions had mean ROI intensities similar to those of the motor and somatosensory cortices and contained layers with different levels of staining (Figure S2).

A moderately low level of IGF-2R intensity/cell was found in the nucleus accumbens, pallidum, hypothalamus, substantia nigra, and deep cerebellar nuclei (DCN). A low level of intensity/cell was observed in the caudoputamen, superior and inferior colliculi, midbrain reticular nucleus, parvicellular reticular nucleus in the medulla, stratum lacunosum moleculare (SLM) in the hippocampus, and the CC (Figure 1D). The lowest levels of IGF-2R were found in fiber tracts, including the CC. In line with previous studies in the rat brain reporting relatively high expression of IGF-2R in choroid plexus [23,25,26], we observed very high levels of staining in the choroid plexus in the lateral and fourth ventricles (Figure 1A), although measurement of relative intensity was highly variable due to irregular tissue folding (Figure S2).

Next, we visually assessed the detailed distribution of IGF-2R within regions (Figures 1A and S2).

#### Olfactory areas:

In both the main and accessory olfactory bulbs, IGF-2R intensity was highest in large cell bodies in the mitral cell layer, where principal glutamatergic output neurons are located (Figure S2). The next highest IGF-2R intensity was observed in the granule and glomerular layers, though overall IGF-2R levels were low in these areas, i.e., their staining intensity was similar to that of the midbrain and caudoputamen. In the glomerular layer, we detected large autofluorescent puncta in both immunostained and secondary-only control sections, which have been reportedly attributed to clusters of lipofuscin granules in microglia [39] (Figure 1A,B). These non-specific signals were excluded from the quantification and description of IGF-2R immunostaining. In the plexiform layers, which contain sparse cell bodies of tufted cells, astrocytes, and inhibitory interneurons, IGF-2R levels were lower than in the granule and glomerular layers, and staining was generally diffuse. Little to no IGF-2R was detected in the olfactory nerve layer, which is composed of unmyelinated axonal fibers. In the anterior olfactory nucleus and piriform cortex, IGF-2R levels were highest in layer II of both regions.

#### Isocortex:

In the six-layer motor, somatosensory, and visual cortices, IGF-2R was detected in neuron perikarya throughout layers II-VI. IGF-2R staining intensity was highest in layer V, where large pyramidal neurons with long-range projections are located, followed by layer II/III (Figure S2). Lower IGF-2R levels were found in layers IV and VI. In layer I, low-intensity diffuse staining similar to that of the SLM in the hippocampus was observed, as well as sparsely distributed stained cell bodies.

#### Hippocampus:

IGF-2R staining in the hippocampus was primarily detected in the SP and SG layers, which contain densely packed neuronal cell bodies, with the highest IGF-2R intensity/cell in the CA2 subregion. In the hilus (polymorphic layer) of the DG, mean IGF-2R intensity/cell was higher than in the SG and CA1 and CA3 SP, due to strong diffuse staining in processes in addition to intense staining in somas (Figures 1D and S2). Sparse cell bodies with intense staining were observed in the SO, SR, and SLM, where mean intensity/cell was low (Figure S2). These areas primarily consist of neuronal processes, glia, and vascular cells, along with sparse inhibitory interneurons. Diffuse staining was also observed with low intensity in the SO, SR, SM, and SLM, and higher intensity in the stratum lucidum (SLu).

#### Striatum and pallidum:

In the striatum, the highest levels of IGF-2R were observed in the olfactory tubercle, where intense staining was found in densely packed cell bodies in layer II and more sparse cell bodies in layer III. Islands of Calleja, found mainly in layer III and composed primarily of granule cells, had relatively low IGF-2R staining (Figure S2). In layer I, low-intensity diffuse staining was observed, similar to layer I of isocortex. Within the pallidum, the highest levels of IGF-2R were found in the magnocellular preoptic nucleus, where cholinergic projection neurons are located [40].

#### Diencephalon:

**Figure 2.**
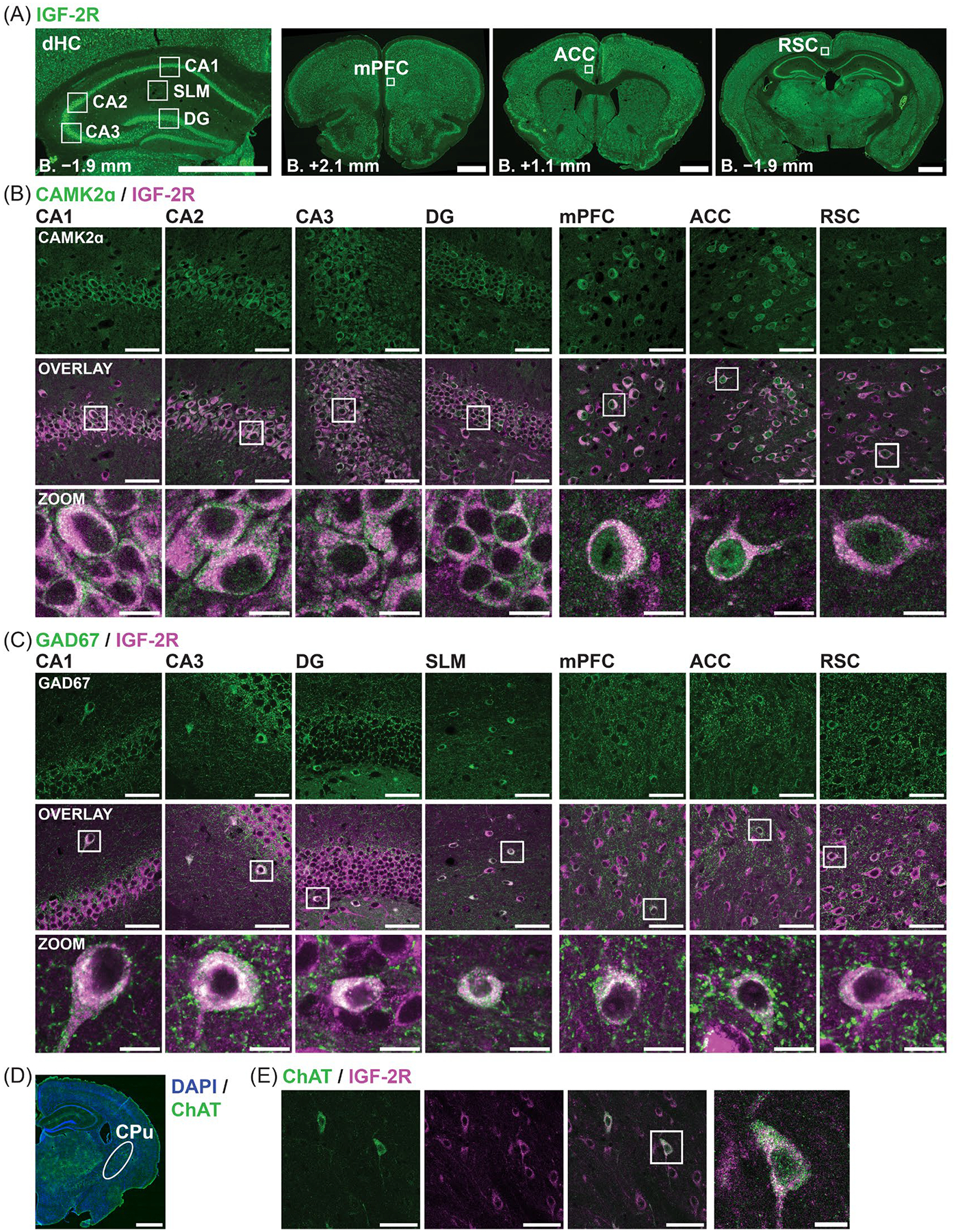
High levels of IGF-2R in excitatory, inhibitory and cholinergic neurons. (**A**) Representative 10× widefield images of IGF-2R immunostaining (green) in coronal mouse brain sections indicating ROIs in subregions of the dorsal hippocampus (dHC; Bregma −1.9 mm) and selected cortical regions important for memory processing: mPFC (Bregma +2.1 mm), ACC (Bregma +1.1 mm), and RSC (Bregma −1.9 mm). Scale bars = 1 mm. (**B**) Representative 63× confocal images of immunofluorescent double stainings of IGF-2R (magenta) and CAMK2α (green) in dHC subregions and cortical regions, showing single stainings of CAMK2α, overlays of IGF-2R and CAMK2α (scale bars = 50 μm), and enlargements of selected areas showing double-positive cells (scale bars = 10 μm). White boxes indicate the areas shown in the zoomed images. (**C**) As (**B**) for costaining of IGF-2R (magenta) and GAD67 (green). (**D**) Representative 10× widefield image of a mouse brain section displaying DAPI (blue) and ChAT (green) staining, indicating location of cholinergic neurons in the caudoputamen (CPu) (scale bar = 1 mm). (**E**) Representative 63× confocal single channel and overlay images for immunofluorescent double staining of IGF-2R (magenta) and ChAT (green) in the CPu (scale bar = 50 μm) with enlarged double-positive cell (scale bar = 10 μm). mPFC—Medial Prefrontal Cortex; ACC—Anterior Cingulate Cortex; B.—Bregma; RSC—Retrosplenial Cortex; dHC—Dorsal Hippocampus; CA1–3—Cornu Ammonis 1–3; DG—Dentate Gyrus; SLM—Stratum Lacunosum Moleculare.

In the thalamus, the highest IGF-2R levels within our sagittal sections were found in the ventral and lateral groups of thalamic nuclei compared to the anterior and reticular nuclei. Previous studies have shown that these groups of thalamic nuclei have different gene expression profiles and functions, with the ventral and lateral nuclei being primarily involved in the relay of sensory and motor information, while the anterior nuclei are associated with limbic circuitry, and the reticular nucleus modulates other thalamic nuclei via inhibitory local projections [41,42]. In the ventral and lateral thalamic nuclei, including the posterior and ventral posteromedial nuclei, levels of diffuse staining were high relative to other brain regions, as reflected by high mean intensity/cell (Figures 1D and S2). In the anterior and reticular thalamic nuclei, IGF-2R levels were similar to those in the hypothalamus, where intensity/cell was moderately low to low. Immunostaining of IGF-2R in coronal sections (Figure 2A) also revealed intense staining in the medial habenula, which contains cholinergic neurons [43], and relatively low levels (similar to the hypothalamus) in the lateral habenula and medially localized thalamic nuclei, which are primarily connected to the limbic system [42].

#### Midbrain:

IGF-2R levels were relatively low in the midbrain, with the highest intensity/cell found in the substantia nigra and the lowest in the midbrain reticular nucleus (Figures 1D and S2). In the superior and inferior colliculi, where the mean intensity/cell was low (Figure 1D), cells with variable IGF-2R intensity were observed, including sparse cells with high levels of staining (Figure S2).

#### Hindbrain:

Within the pons and medulla, IGF-2R levels were highest in the facial motor nucleus, which is composed of the cell bodies of cholinergic lower motor neurons of cranial nerve VII. Intense IGF-2R staining was observed throughout the large somas of these cells as well as in many distinct individual processes (Figure S2). Low IGF-2R intensity/cell was found in the parvicellular reticular nucleus, with similar levels observed in the medullary, intermediate and pontine reticular nuclei, and the superior olivary complex. Intermediate IGF-2R levels within the hindbrain were found in the vestibular nuclei, cuneate nucleus, lateral reticular nucleus, nucleus ambiguus, and pontine grey.

#### Cerebellum:

In the cerebellar cortex, IGF-2R levels were highest in the Purkinje cell layer—composed of large, highly branched inhibitory output neurons—followed by the granular layer (Figure S2). Low intensity diffuse staining was observed in the molecular layer, similar to cortical layer I and the SLM, while staining in the arbor vitae (fiber tracts) was nearly undetectable. In the DCN, which contains glutamatergic projection neurons and inhibitory interneurons, IGF-2R intensity was variable among cells and replicate sections (Figure 1D).

In summary, these data indicate that across all brain regions, IGF-2R is primarily enriched in neuronal cell bodies, particularly in large projection neurons, including pyramidal neurons in the hippocampus and cortical layer V, cerebellar Purkinje cells, and olfactory bulb mitral cells. High IGF-2R intensity was also observed in areas containing cholinergic neuron somas: the facial motor nucleus, medial habenula, and magnocellular preoptic nucleus. IGF-2R levels were also high in the choroid plexus, but low in most other brain areas containing few to no neuronal cell bodies. Very low to undetectable levels of IGF-2R immunostaining were found in fiber tracts. However, areas composed mainly of dendritic arbors—such as cortical layer I, the molecular layer of the cerebellar cortex, and hippocampal subregions—had low levels of diffuse staining.

### 3.2. Comparison of the Regional Distribution of IGF-2R and CD-M6PR in the Mouse Brain

To compare the CD-M6PR and IGF-2R distributions in the mouse brain, a recombinant monoclonal antibody to CD-M6PR was used for immunostaining in parallel to IGF-2R (Figure 1E). The brain CD-M6PR staining distribution was classified according to quartiles, i.e., high (75–100th), moderately high (50–75th), moderately low (25–50th), and low (0–25th), with the 0, 25, 50, 75 and 100th percentiles, respectively, corresponding to fluorescence intensity per cell values (AU) of 0.235, 0.799, 1.046, 1.309, and 3.319 (Figure 1F; N = 3).

Similar distributions of the two receptors were found in multiple brain regions (Figure 1). High to moderately high intensity/cell in the somatosensory and motor cortices was observed for both receptors, with the highest levels in layer V. In the hippocampus, both receptors were mainly detected in the SP and SG layers, with the highest levels in CA2 and sparse immunostained cell bodies in SO, SR, and SLM. Within olfactory regions, the highest levels of both proteins were found in the mitral cell layer of the olfactory bulb and layer II of the anterior olfactory nucleus. In the hindbrain, both had low intensity/cell in the parvicellular reticular nucleus, and higher levels in the facial motor nucleus, although the difference was much greater for IGF-2R (Figure 1D,F). Relative intensity/cell for both receptors was low in the midbrain reticular nucleus and moderately low in the DCN. The cerebellar cortex also had similar staining patterns, with levels within the cerebellar cortex highest in the Purkinje cell layer, lowest in the arbor vitae, and intermediate in the granule layer (Figure 1E,F). The lowest levels of both receptors were found in the fiber tracts. The choroid plexus was intensely stained for both receptors.

However, the distributions of IGF-2R and CD-M6PR immunostaining differed in several brain regions. The most prominent differences were observed in the diencephalon: intensity/cell in the hypothalamus was high for CD-M6PR, but moderately low for IGF-2R, whereas intensity/cell in the ventral and lateral thalamic nuclei was high for IGF-2R but low for CD-M6PR. Among thalamic nuclei, CD-M6PR levels in the anterior and reticular groups were higher than in the ventral and lateral groups—the inverse of the distribution of IGF-2R. Substantially different distributions were also found in the olfactory bulb, where CD-M6PR immunostaining was very low to absent in the glomerular, granule cell and plexiform layers. Within the isocortex, a relatively high intensity of CD-M6PR but not IGF-2R was observed in layer VIb, a densely packed band of diverse types of primarily excitatory neurons (reviewed in [44]). In the hippocampus, CD-M6PR levels were notably higher in the SP of CA3 compared to CA1, whereas IGF-2R intensity/cell was similar in these subregions (Figure 1D,F). The diffuse staining observed in the hilus and SLu for IGF-2R was not detected for CD-M6PR. CD-M6PR levels in the subiculum were also relatively lower than those of IGF-2R. Compared to the distribution of IGF-2R, levels of CD-M6PR were relatively higher in the midbrain and relatively lower in the striatum. Levels of diffuse staining in the caudoputamen were also lower for CD-M6PR (Figure 1A,E). Furthermore, levels of CD-M6PR in layer I of the isocortex, SO, SR, SM, and SLM of the hippocampus, and the molecular layer of the cerebellar cortex were very low, similar to levels in fiber tracts, whereas IGF-2R immunostaining in these regions was higher than in fiber tracts.

### 3.3. IGF-2R Is Abundant in Hippocampal, Cortical and Cholinergic Neurons

We next characterized the distribution of IGF-2R protein across brain cell types by conducting a series of double immunofluorescent stainings in coronal sections using antibodies to IGF-2R and cell-type-specific markers. As IGF-2R is required for memory formation [2], we focused on brain regions important for learning and memory, specifically the dHC subregions CA1, CA3, and DG, and the mPFC, ACC, and RSC (Figure 2A). In dHC sections, we also analyzed the IGF-2R distribution in cholinergic neuron populations.

We first examined the distribution of IGF-2R in neuronal populations using the following markers: calcium/calmodulin-dependent protein kinase II subunit alpha (CaMK2α) for excitatory neurons, glutamate decarboxylase 67 (GAD67) for inhibitory neurons, and choline acetyltransferase (ChAT) for cholinergic neurons. IGF-2R was consistently detected at high levels in nearly all these neurons (Figure 2), in line with findings based on morphological identification of cell types. In fact, across all images analyzed, we did not observe any of these neurons that were negative for IGF-2R staining.

IGF-2R signal in the hippocampus and cortical areas was strongly detected in CaMK2α-expressing cells, in agreement with our previous report [2]. The highest levels of IGF-2R within CaMK2α+ neurons were found in the perinuclear region, consistent with reported localization of the receptor in the TGN [45]. Robust staining was also observed consistently in somas and frequently in the proximal dendrites, particularly the apical dendrites of hippocampal and cortical pyramidal neurons (Figures 2B and S3). No staining was detected in nuclei.

In GAD67+ inhibitory interneurons, IGF-2R was strongly expressed in all regions analyzed, including the hilus and SLM (Figures 2C and S3). The intracellular distribution of IGF-2R was similar to that found in CaMK2α+ excitatory neurons, with the strongest immunostaining in the perinuclear region and high levels throughout interneuron somas. IGF-2R was also observed in the proximal processes of interneurons in all subregions of the dHC and the three cortical regions.

Cholinergic neurons labelled with anti-ChAT were found in the caudoputamen in the dorsal striatum but not detected in the dHC (Figure 2D), consistent with previous reports [46–48]. Striatal ChAT+ cells had high levels of IGF-2R throughout the soma as well as in some proximal processes (Figure 2E). Some distal ChAT+ processes containing IGF-2R were also observed (Figure 2E).

**Figure 3.**
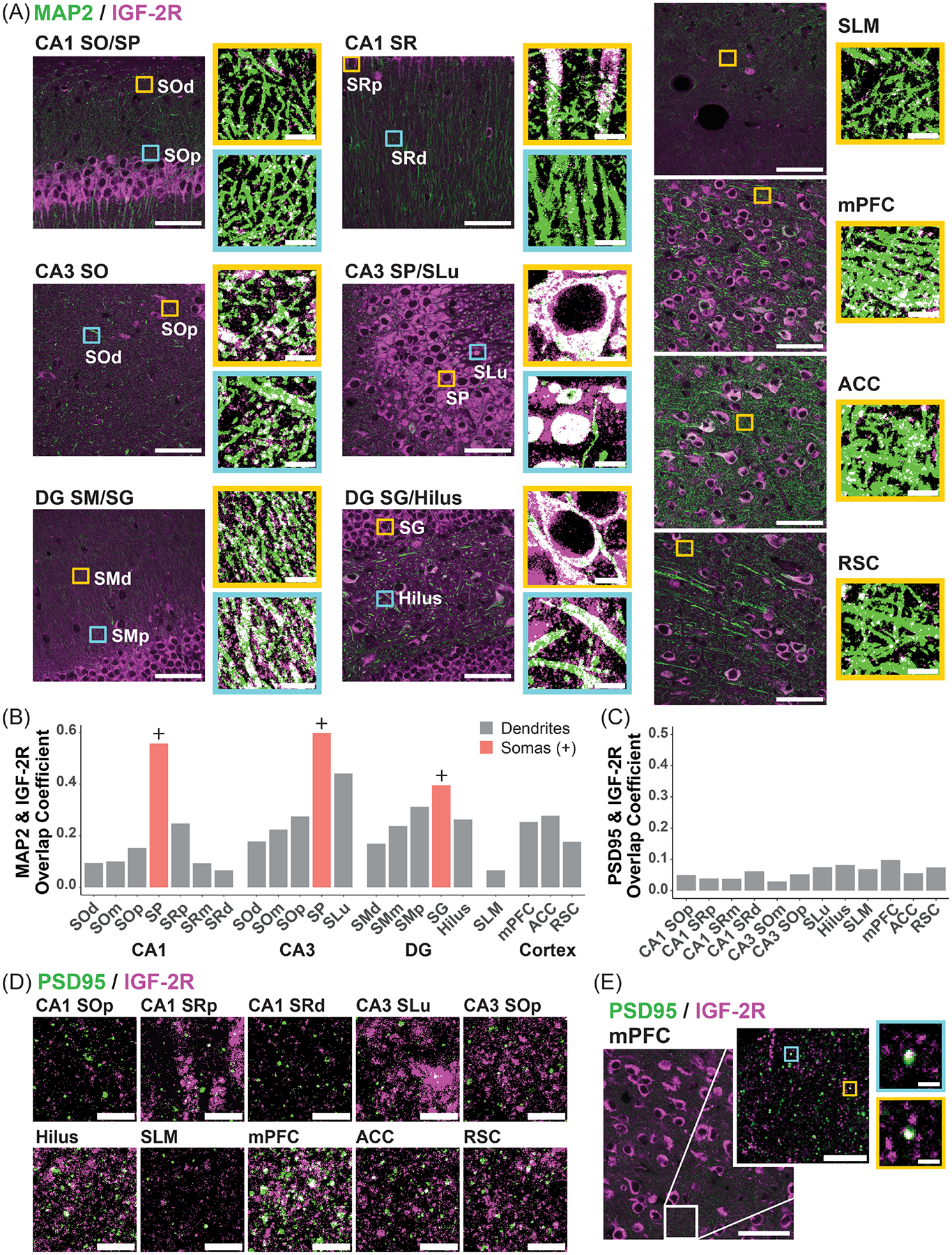
IGF-2R is detected in proximal and distal neuronal processes. (**A**) High-resolution 63× confocal images of immunofluorescent double staining of MAP2 (green) and IGF-2R (magenta) in the dHC, mPFC, ACC, and RSC (scale bar = 50 μm). Enlarged binary overlays are shown for selected small areas containing MAP2+ dendrites or neuronal cell bodies, indicated by colored boxes, as representative examples of the colocalization of signals (in white) (scale bars = 5 μm). (**B**) Barplot of Mander’s overlap coefficients (MOCs) indicating degree of colocalization of MAP2 and IGF-2R in dendrites in dHC subregions and cortices (N = 1). MOCs for hippocampal neuron soma layers (SP and SG) are included as positive controls (+ red bars). (**C**) Barplot of MOCs for PSD95 and IGF-2R in dHC subregions and cortices, excluding neuron somas (N = 1). (**D**) Binary overlays of IGF-2R (magenta) and PSD95 (green) double staining for small representative areas within ROIs (scale bars = 5 μm). (**E**) High-resolution 63× confocal image of immunofluorescent double staining of PSD95 and IGF-2R in the mPFC (scale bar = 50 μm), showing enlargement of a small area of staining outside neuronal cell bodies (scale bar = 10 μm) and further enlargements of two double-positive puncta (scale bar = 1 μm). CA1–3—Cornu Ammonis 1–3; DG—Dentate Gyrus; SO—Stratum Oriens; SP—Stratum Pyramidale; SR—Stratum Radiatum; SLu—Stratum Lucidum; SLM—Stratum Lacunosum Moleculare; SM—Stratum Moleculare; SG—Stratum Granulosum; p—Proximal; m—Medial; d—Distal; mPFC—Medial Prefrontal Cortex; ACC—Anterior Cingulate Cortex; RSC—Retrosplenial Cortex.

To determine the distribution of IGF-2R in dendrites, we next analyzed colocalization of IGF-2R and microtubule-associated protein 2 (MAP2) immunostaining in high-resolution images of dHC subregions and cortical regions (Figure 3A,B; N = 1 per region). Colocalization was assessed using Mander’s overlap coefficient (MOC), which represents the colocalized intensity as a proportion of total fluorescent intensity following background subtraction [49,50]. The SP and SG ROIs, which contain mainly somas, were used as positive controls to assess relative MAP2/IGF-2R colocalization levels. The overlap of MAP2 and IGF-2R within subregions is depicted in enlarged representative examples of binary overlays (Figure 3A). The strongest colocalization of IGF-2R and MAP2 in dendrites was found in the CA3 SLu, with an MOC of 0.44, as compared to MOCs in neuron somas of 0.55–0.60 in the SP of CA1 and CA3 and 0.40 in the SG (Figure 3B). In the SLu, strong IGF-2R immunostaining was observed both in the MAP2+ proximal dendrites of CA3 pyramidal neurons and in the surrounding area (Figure 3A), suggesting that IGF-2R is also abundant in mossy fibers—axonal tracts that do not contain MAP2—or their terminals [51]. Strong IGF-2R immunostaining surrounding and overlapping MAP2+ processes was similarly observed in the hilus (Figure 3A), where mossy fibers terminate on hilar mossy cells.

Moderate colocalization (MOC 0.22–0.32) of IGF-2R and MAP2 was observed in the proximal and medial CA3 SO and DG SM, proximal CA1 SR, hilus, mPFC, and ACC (Figure 3B). Low colocalization (MOC 0.15–0.18) was observed in the distal CA3 SO and DG SM, proximal CA1 SO, and the RSC. Very low colocalization (MOC 0.06–1.0) was found in the SLM and medial and distal CA1 SO and SR. In all dHC subregions, colocalization of IGF-2R and MAP2 was highest proximal to neuronal somas and lowest at distal sites (Figure 3A,B). However, the distribution differed: within CA1 SR, IGF-2R was primarily concentrated in the proximal apical dendrites within 10 μm of pyramidal neuron somas, whereas the DG SM and CA3 SO showed a more gradual reduction in dendritic IGF-2R levels with increasing distance from neuronal cell body layers.

To determine whether IGF-2R is present at dendritic spines, we next analyzed colocalization of IGF-2R and postsynaptic density protein 95 (PSD95) in the hippocampal and cortical ROIs, excluding neuron somas. Colocalization of PSD95 and IGF-2R was very low across all quantified regions (MOC 0.029–0.098; Figure 3C,D), with only sparse IGF-2R+ spines observed (shown for mPFC; Figure 3E).

In summary, IGF-2R was consistently and robustly expressed in excitatory and inhibitory neurons in the hippocampus and cortical regions important for memory and in striatal cholinergic neurons. IGF-2R was predominantly localized in perikarya and proximal apical dendrites of hippocampal and cortical pyramidal neurons. IGF-2R was also detected in more distal dendrites, particularly in CA3 SO, DG SM, and striatal cholinergic neurons. IGF-2R rarely colocalized with PSD95. We also observed strong IGF-2R immunostaining in the hilus and SLu, which, based on its distribution, appeared to be localized in mossy fibers and/or their giant terminals.

### 3.4. Minimal-to-Undetectable Immunostaining of IGF-2R in Astrocytes and Microglia

To determine IGF-2R levels in astrocytes and microglia, we used antibodies to glial fibrillary acidic protein (GFAP) and aldehyde dehydrogenase family 1 member L1 (ALDH1L1) to identify astrocytes, and to ionized calcium-binding adaptor molecule 1 (IBA1) to identify microglia.

**Figure 4.**
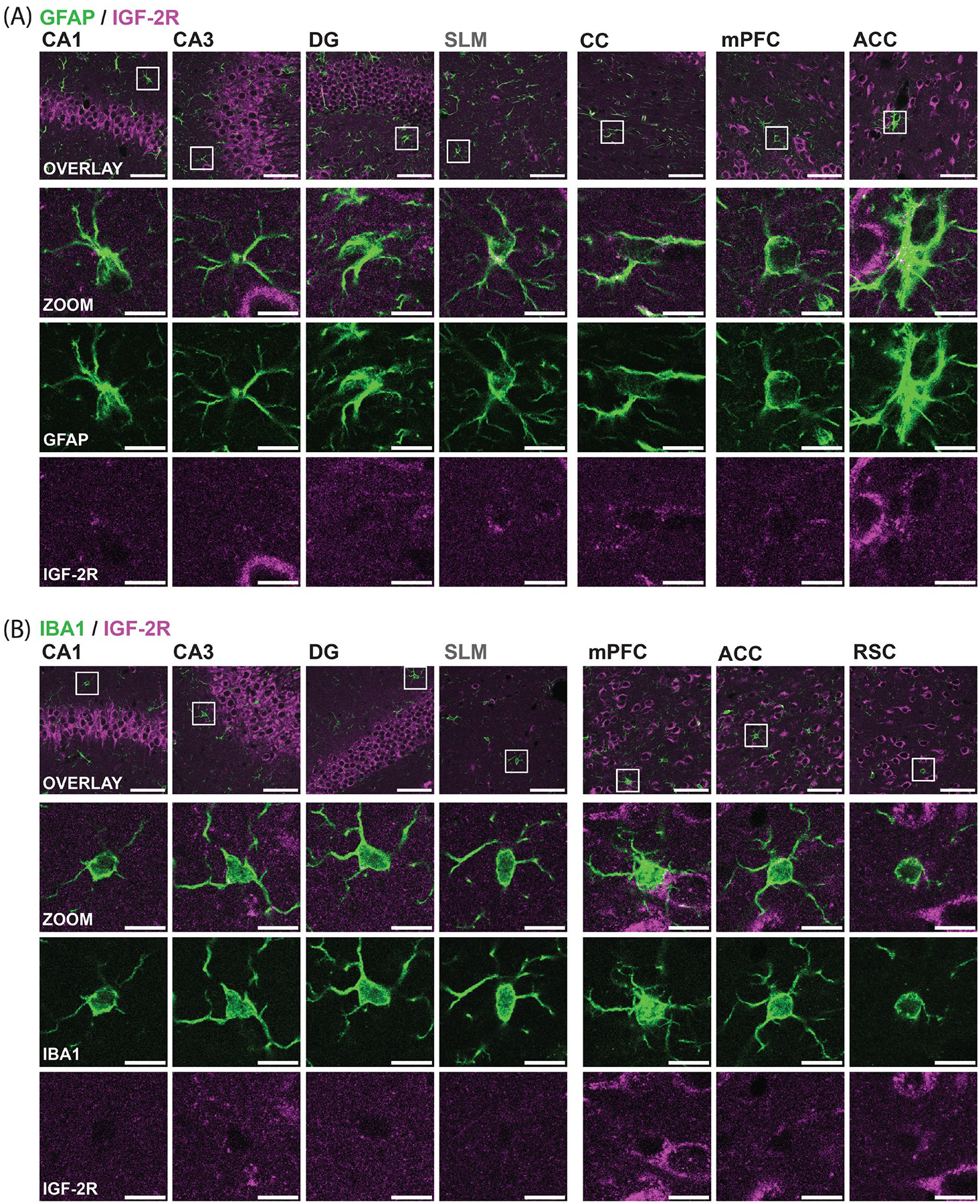
Minimal to undetectable levels of IGF-2R immunostaining in astrocytes and microglia. (**A**) Representative 63× confocal image overlays of immunofluorescent double staining of IGF-2R (magenta) and GFAP (green) in dorsal hippocampus subregions, corpus callosum (CC), mPFC, and ACC (scale bars = 50 μm) with enlargements of selected areas showing single channel and overlay images (scale bars = 10 μm). White boxes indicate the areas shown in the zoomed images. (**B**) As (**A**) for IGF-2R (magenta) and IBA1 (green) costaining. CA1–3—Cornu Ammonis 1–3; DG—Dentate Gyrus; SLM—Stratum Lacunosum Moleculare; mPFC—Medial Prefrontal Cortex; ACC—Anterior Cingulate Cortex; RSC—Retrosplenial Cortex.

GFAP+ astrocytes showed little to no detectable IGF-2R staining in the CA1, CA3, and DG subregions of the hippocampus, as well as in the mPFC and ACC (Figure 4A), consistent with previous research [2,23,25]. Small puncta were observed within astrocyte cell bodies and processes, but they were also present in the background staining, indicating non-specific detections. As GFAP+ cells were not detected in the RSC and were sparse in the mPFC and ACC, an additional marker more widely expressed within cortical astrocytes, ALDH1L1 [52], was used, which confirmed minimal IGF-2R staining within astrocytes (Figure S4). However, in the SLM and CC, IGF-2R was consistently detected at low levels in GFAP+ and ALDH1L1+ cells, primarily in the perinuclear area (Figures 4A and S4). This low, but detectable, expression level may result from distinct astrocyte subpopulations in the SLM and CC expressing relatively higher levels of IGF-2R [53–55] or from more efficient differential detection due to lower levels of diffuse staining in overlapping neuronal processes.

IBA1+ microglia in the hippocampus and cortical regions exhibited undetectable IGF-2R immunostaining (Figure 4B). Autofluorescent puncta with overlapping signal between channels stained for IGF-2R and IBA1 were observed within microglia, as recently reported [56]. This staining, however, was non-specific, as confirmed in images of an unstained channel (647 nm) and in control sections stained in parallel without anti-IGF-2R. Enlarged confocal images of microglia shown in Figure 4B were selected from planes not containing autofluorescent puncta.

In summary, little to no IGF-2R was observed in astrocytes and microglia.

### 3.5. IGF-2R Is Detected at Moderate Levels in a Subset of Oligodendrocyte Lineage Cells

Oligodendrocytes were identified using an antibody to SRY-box transcription factor 10 (SOX10), which is specifically expressed in the nuclei of oligodendrocyte lineage cells, including OPCs [57].

**Figure 5.**
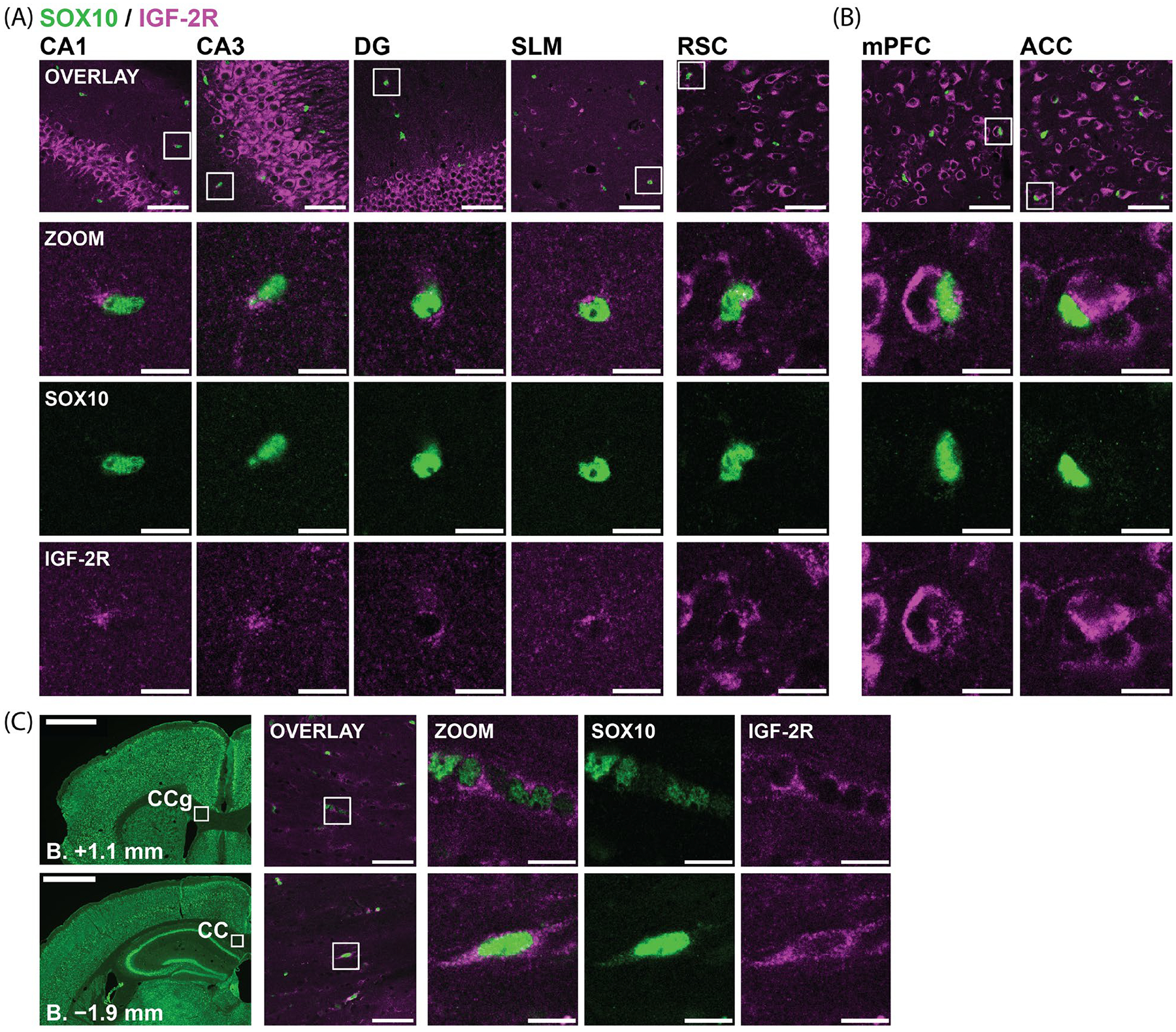
IGF-2R is found in oligodendrocyte lineage cells in the hippocampus and corpus callosum. (**A**) Representative 63× confocal images of double immunofluorescent staining of IGF-2R (magenta) and SOX10 (green) in dorsal hippocampus subregions and RSC (scale bars = 50 μm) with enlargements of selected areas showing double positive cells (scale bars = 10 μm). White boxes indicate the areas shown in the zoomed images. (**B**) Representative images of IGF-2R and SOX10 staining in mPFC and ACC with enlargements of areas showing satellite oligodendrocytes (green) overlapping IGF-2R+ neuron somas (magenta). (**C**) ROIs in the genu (g) and body of the corpus callosum (CC) indicated in 10× widefield images of sections stained for IGF-2R (Bregma +1.1 mm and −1.9 mm, respectively; scale bars = 1 mm), with representative 63× confocal images and enlargements of selected areas showing double-positive cells shown for each ROI. CA1–3—Cornu Ammonis 1–3; DG—Dentate Gyrus; SLM—Stratum Lacunosum Moleculare; RSC—Retrosplenial Cortex; mPFC—Medial Prefrontal Cortex; ACC—Anterior Cingulate Cortex; B.—Bregma.

In the dHC, variable levels of IGF-2R immunostaining were observed surrounding SOX10+ nuclei. Perinuclear IGF-2R was detected in approximately half of the SOX10+ nuclei in the SO, SR, SM, and SLM (Figure 5A), with the remainder exhibiting ambiguous or undetectable immunostaining. Unlike the distribution of IGF-2R in neurons, IGF-2R immunostaining surrounding SOX10+ nuclei in the dHC was primarily concentrated in a small area on one side of the nucleus, consistent with localization in the Golgi apparatus and TGN [45]. In the hilus, SLu, SG, and SP, assessment of IGF-2R immunostaining in SOX10+ cells was ambiguous due to overlap with strongly stained neuronal somas and/or processes. IGF-2R staining surrounding the majority of SOX10+ nuclei observed in cortical regions was also unclear, as these nuclei were located in very close proximity to neuron somas, a defining characteristic of satellite oligodendrocytes [58] (Figure 5B). In the RSC, but not in the ACC or mPFC, we observed some SOX10+ nuclei that did not appear to overlap with cortical neuron cell bodies and had perinuclear IGF-2R staining similar to that described in the dHC (Figure 5A).

IGF-2R immunostaining was also analyzed in the CC adjacent to the dHC, and in the anterior CC, also known as the genu, adjacent to the ACC (Figure 5C). In these areas, low to moderate levels of IGF-2R were observed surrounding the majority of SOX10+ nuclei. Some of these cells were clustered in rows (Figure 5C), a formation characteristic of myelinating oligodendrocytes [59,60]. We also found isolated SOX10+ cells with elongated nuclei in which IGF-2R staining surrounded the entire nucleus and extended into their proximal processes at both ends (Figure 5C).

In summary, perinuclear IGF-2R staining was detected in a subset of oligodendrocyte lineage cells in the dHC and RSC, as well as in most SOX10+ cells in the CC. The IGF-2R level in these cells was lower than in hippocampal and cortical neurons.

### 3.6. Moderate Levels of IGF-2R Are Detected in Mural Cells of the Vasculature

Co-staining of IGF-2R with antibodies to two markers of vascular cells was performed: CD31 (also called platelet endothelial cell adhesion molecule 1, or PECAM1), a transmembrane receptor expressed by vascular endothelial cells and certain immune cells that forms intercellular junctions between endothelial cells [61], and platelet-derived growth factor receptor beta (PDGFRβ), a cell surface receptor expressed in mural cells and some fibroblasts [36,62,63]. Mural cells collectively refer to pericytes, which surround endothelial cells at capillaries, and vascular smooth muscle cells (VSMCs) found at larger blood vessels [64].

**Figure 6.**
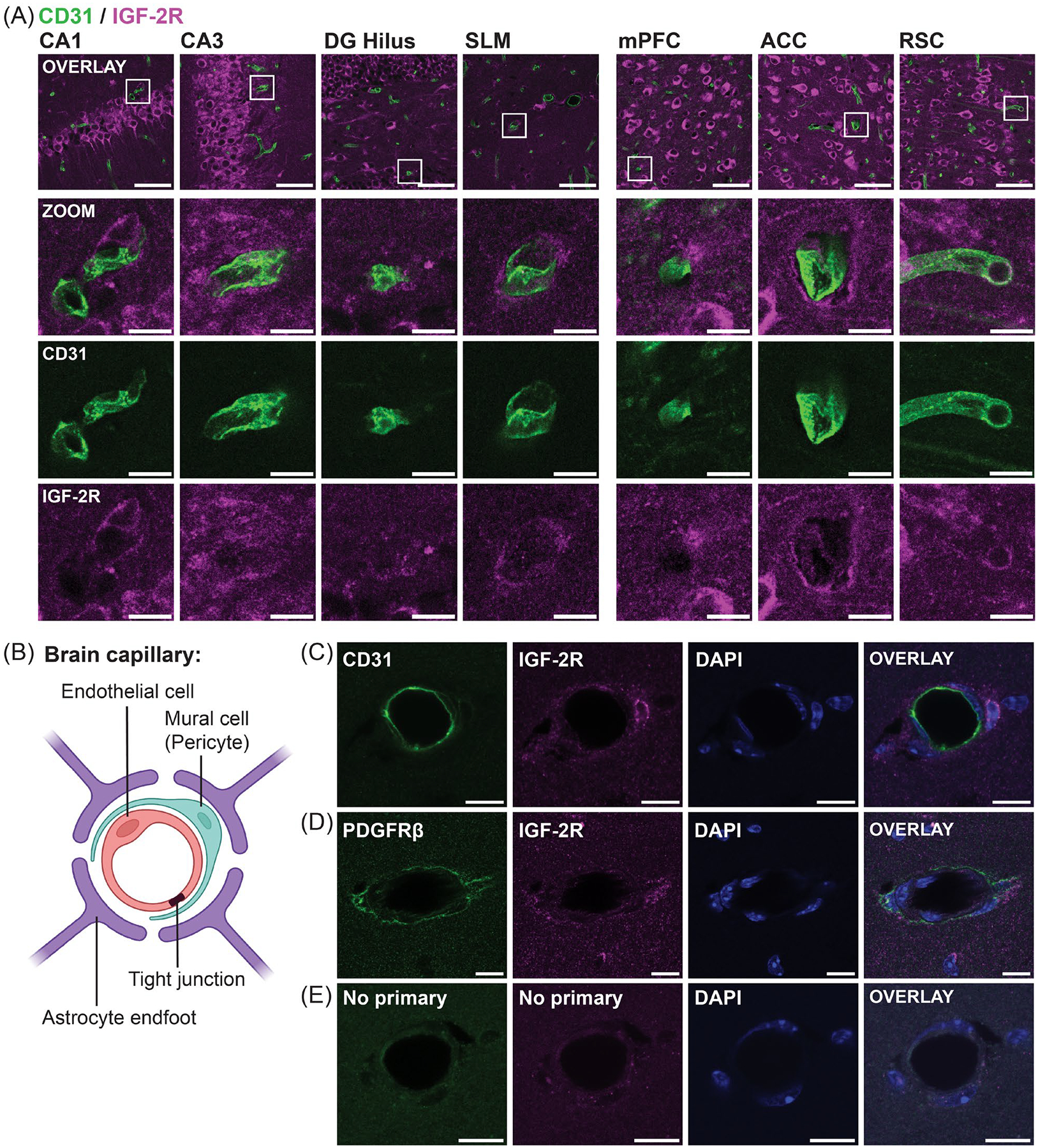
IGF-2R is detected in mural cells but not endothelial cells of the brain vasculature. (**A**) Representative 63× confocal images of double immunofluorescent staining of IGF-2R (magenta) and CD31 (green) in dorsal hippocampus subregions, mPFC, ACC, and RSC (scale bars = 50 μm) with enlargements of areas showing vasculature with adjacent IGF-2R and CD31 staining (scale bars = 10 μm). White boxes indicate the areas shown in the zoomed images. (**B**) Schematic of a brain capillary. Created in BioRender https://BioRender.com/41fg87q (accessed on 17 March 2025). (**C**) Representative high-resolution 63× confocal images of double immunofluorescent staining of IGF-2R and CD31 with DAPI showing a blood vessel in the SLM (scale bars = 10 μm). (**D**) As (**C**) for IGF-2R and PDGFRβ. (**E**) Control staining conducted in parallel with (**C**,**D**) without the primary antibody. CA1–3—Cornu Ammonis 1–3; DG—Dentate Gyrus; SLM—Stratum Lacunosum Moleculare; mPFC—Medial Prefrontal Cortex; ACC—Anterior Cingulate Cortex; RSC—Retrosplenial Cortex.

Little overlap was observed between IGF-2R and CD31 immunostaining (Figure 6A). However, IGF-2R staining of low to moderate intensity was consistently detected in neurovascular structures adjacent to CD31 throughout the dHC and cortical regions, often encircling CD31 staining (Figure 6A). We next examined colocalization of IGF-2R with CD31 and PDGFRβ at small blood vessels in the SLM identified by lumens surrounded by three to five DAPI+ nuclei [65] (Figure 6B). IGF-2R was not detected in the somas of CD31+ cells in the innermost ring around blood vessel lumens (Figure 6C) but was found in surrounding PDGFRβ+ mural cells (Figure 6D). The specificity of staining was confirmed in sections stained in parallel without the primary antibody (Figure 6E).

In summary, IGF-2R was detected in mural cells, albeit at a lower level compared to neurons.

### 3.7. Multiplex Immunostaining Confirms the Relative Abundance of IGF-2R Across Neuronal,Glial and Vascular Cell Types

To obtain a profile of IGF-2R abundance across cell types in the dHC and cortical regions, we next performed a multiplex tyramide signal amplification (TSA) assay, a method that amplifies weak immunoreactivity signals [66–68].

**Figure 7.**
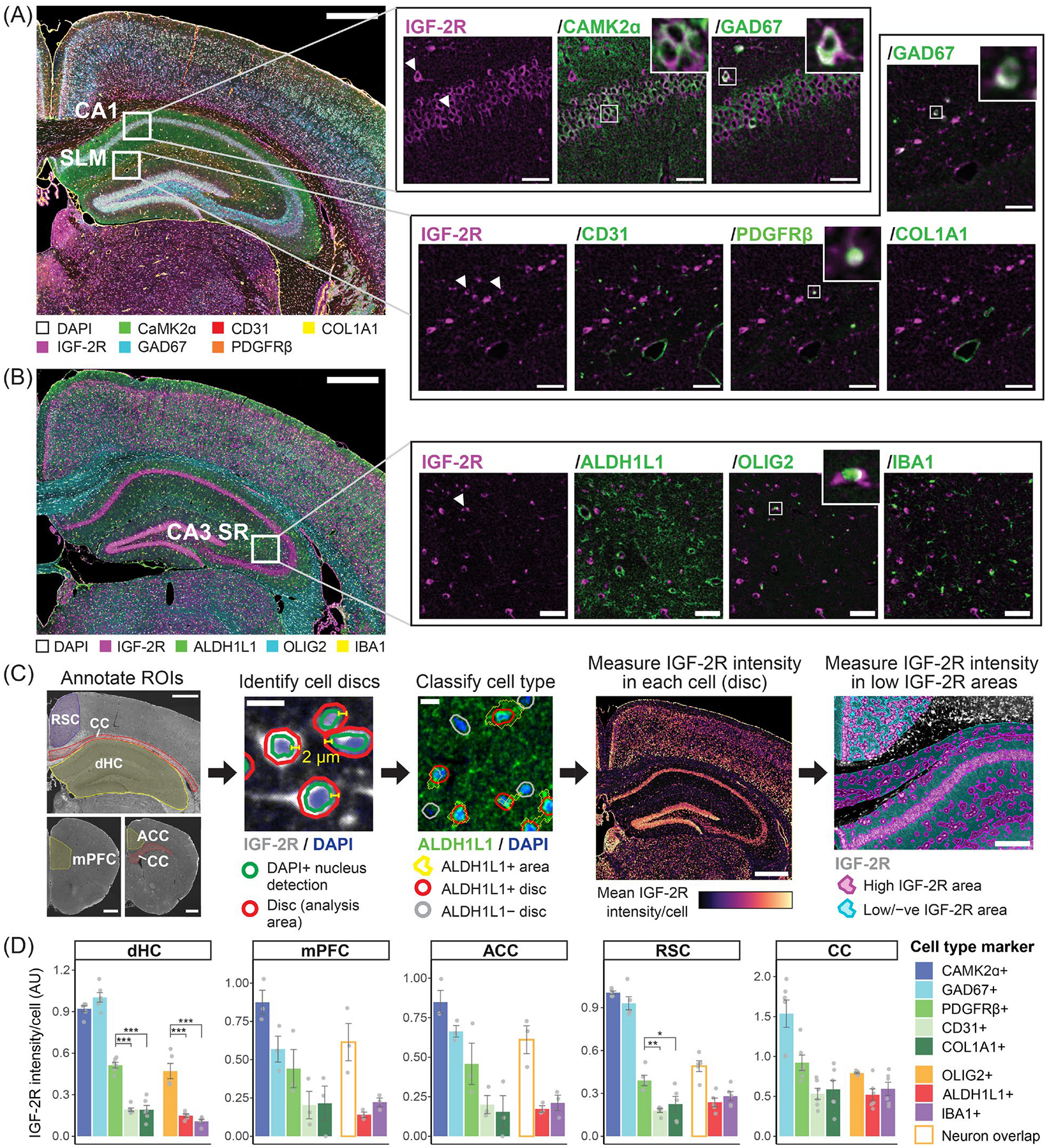
Multiplex quantification of relative IGF-2R levels in different brain cell types. (**A**) Representative 20× fluorescent photomicrographs of multiple-label tyramide signal amplification (TSA) immunostaining for IGF-2R and specific markers of neuronal and vascular cell types in dorsal hippocampus (dHC) and surrounding cortex. Left: 7-channel overlay including DAPI (scale bar = 500 μm). Right: Enlargements of selected subregions showing IGF-2R staining and overlay with individual cell type markers. Areas showing selected double-positive cells are enlarged—indicated by white arrows in IGF-2R images and white boxes in overlays. Enlargements are not shown for cell populations with low levels of IGF-2R. Scale bars = 50 μm. (**B**) As (**A**) for IGF-2R and markers of glial cell types, with 5-channel overlay. (**C**) Schematic of the method for quantification of IGF-2R levels in brain cell populations. In brief, first ROIs were manually annotated (scale bars = 500 μm) and DAPI+ nuclei were identified (scale bar = 10 μm). The quantification area (‘disc’) for each cell comprised the nucleus and a 2 μm width border around the nucleus. Cell types were classified using supervised machine learning algorithms (scale bar = 10 μm). Mean IGF-2R intensity was then measured in each disc (arbitrary units [AU]; scale bar = 500 μm). Finally, to account for diffuse IGF-2R staining in neuronal processes, areas with low/negative IGF-2R levels in each ROI were identified (scale bar = 200 μm) and median IGF-2R intensity of these areas was subtracted from intensity/cell measurements. (**D**) Mean IGF-2R intensity/cell for each cell type marker in the dHC (N = 5–6), mPFC (N = 3), ACC (N = 3), RSC (N = 5), and corpus callosum/alveus (CC) (N = 6). Data are presented as mean ± SEM and points correspond to independent subject means. Differences in IGF-2R levels between markers were analyzed within each ROI using one-way ANOVAs with Tukey’s post hoc tests. A significant main effect of marker was observed in all regions (*p* < 0.001), and significant results of selected post hoc tests are indicated (* *p* < 0.05, ** *p* < 0.01, *** *p* < 0.001; Table S1). Mean intensities of OLIG2+ populations in cortical regions were spuriously high due to overlap with neuronal cell bodies (see Figure S1).

Two sets (panels) of antibodies were used: (1) IGF-2R with the neuronal markers CaMK2α and GAD67, and vascular markers CD31, PDGFRβ, and collagen type I alpha 1 chain (COL1A1; Figure 7A), and (2) IGF-2R with the glial markers ALDH1L1, OLIG2, and IBA1 (Figure 7B). COL1A1 was used as a marker of central nervous system (CNS) fibroblasts, which include subtypes found in perivascular spaces, meninges, and the choroid plexus [63,69]. The transcription factor OLIG2, which is localized to the nucleus, was used as a marker of oligodendrocyte lineage cells [70].

Relative mean IGF-2R intensity/cell was quantified in cell populations in the dHC, mPFC, ACC, RSC and CC by the following method (Figure 7C; Methods). Cells were identified by DAPI staining, and a 2 μm perinuclear area (hereafter referred to as a disc) was demarcated for quantifications to capture the area where IGF-2R is primarily localized. Discs were then classified according to cell type-specific marker expression. For IBA1+ cells, in order to reduce the confounding signal of overlapping neurons, quantification was repeated using IBA1 staining itself to identify the quantification area (Figure S1).

In the dHC, the quantifications confirmed that the highest levels of IGF-2R are detected in CaMK2α+ and GAD67+ neuronal populations, which both had significantly higher IGF-2R levels than all vascular and glial populations (*p* < 0.001; N = 5–6; Figure 7D; Table S1). The next highest levels of IGF-2R in the dHC were found in PDGFRβ+ and OLIG2+ populations and were significantly higher than in all other glial and vascular populations studied (*p* < 0.001; N = 5–6 subjects), consistent with our confocal double staining experiments. Mean normalized IGF-2R intensity in both PDGFRβ+ and OLIG2+ discs was approximately half that observed in neuronal discs, although our analysis may underestimate IGF-2R levels in neuron somas due to differences in cell size and shape, in addition to the possibility of unequal amplification of signals by TSA [71]. No significant differences in IGF-2R levels were observed among the CD31+, COL1A1+, ALDH1L1+, and IBA1+ populations. In discs positive for two or more vascular markers, IGF-2R intensity was similar to that of CD31+ and COL1A1+ populations and significantly lower than in PDGFRβ+ discs (Figure S5).

We also analyzed differences in IGF-2R levels among glial and vascular populations in dHC subregions using a two-way ANOVA (Figure S5; Table S2). We found significant main effects of marker (F(5, 155) = 54.753, *p* < 0.001) and subregion (F(5, 155) = 4.081, *p* = 0.00165) and an interaction effect (F(25, 155) = 1.731, *p* = 0.0235). IGF-2R intensity in OLIG2+ discs was significantly higher compared to both ALDH1L1+ and IBA1+ discs in the SO and SR of both CA1 and CA3, and to IBA1+ discs in the DG SM and SLM (*p* < 0.05; N = 5). No significant differences in IGF-2R levels between dHC subregions were found for any of the three glial markers. Thus, OLIG2+ cells with high levels of IGF-2R compared to other glial cell populations were observed throughout the dHC. Among vascular populations, mean IGF-2R intensity was significantly higher in PDGFRβ+ discs than both CD31+ and COL1A1+ discs in the CA1 SO, CA1 SR, and CA3 SO subregions, but not in the CA3 SR, DG SM, and SLM (*p* < 0.05; N = 6; Figure S5; Table S2). Moreover, IGF-2R levels in PDGFRβ+ discs were significantly higher in the CA1 SO subregion compared to the CA3 SR (*p* < 0.001), SM (*p* = 0.0064), and SLM (*p* < 0.001), whereas IGF-2R levels in CD31+ and COL1A1+ discs were not significantly different across subregions (Figure S5; Table S2). As the CA1 SO contains a greater proportion of capillaries and smaller diameter vessels compared to the latter three subregions due to its distance from the internal transverse hippocampal artery, the higher levels of IGF-2R in PDGFRβ+ discs in this region may reflect an enrichment of IGF-2R in pericytes, which surround capillaries, compared to VSMCs found at larger vessels [72].

In the three cortical regions, mPFC, ACC and RSC, a similar pattern of IGF-2R abundance across cell types was observed as in the dHC, with the highest levels in neurons, followed by PDGFRβ+ and OLIG2+ populations (Figure 7D). However, as noted in double staining experiments, accurate quantification of IGF-2R in cortical oligodendrocytes was impeded by the overlap of a large proportion of these cells with neuron somas (Figure S1). Consequently, IGF-2R levels in cortical OLIG2+ discs could not be accurately quantified. Among vascular populations, mean IGF-2R intensity in PDGFRβ+ discs was significantly higher than in CD31+ and COL1A1+ discs in the RSC (CD31+: *p* = 0.0040, COL1A1+: *p* = 0.040; N = 5). In the mPFC and ACC, the profile of IGF-2R levels across vascular populations was similar to the RSC, but differences were not significant (N = 3; Figure 7D; Table S1). IGF-2R levels were consistently low in ALDH1L1+, IBA1+, CD31+ and COL1A1+ discs. In addition, we observed that while IGF-2R levels in the RSC were similar in CaMK2α+ and GAD67+ discs, in the mPFC and ACC, they were lower in GAD67+ discs, with a greater difference in the mPFC (Figures 7D and S6). This may reflect differences in IGF-2R-mediated functions among cortical inhibitory neuron populations.

In the CC adjacent to the dHC and ACC, the highest levels of IGF-2R were found in sparse GAD67+ cells, followed by PDGFRβ+ and OLIG2+ discs, as observed in the cortex and hippocampus (Figure 7D). However, differences in IGF-2R intensity among glial and vascular populations were not significant. Furthermore, the raw mean IGF-2R intensity in OLIG2+ discs was significantly lower in the CC than in the dHC (*p* = 0.00260; N = 5; Figure S5; Table S3), supporting the hypothesis that IGF-2R levels differ across populations of oligodendrocytes. No significant difference in IGF-2R levels between the CC and dHC was observed for ALDH1L1+ and IBA1+ populations (Figure S5).

In summary, quantification of multiplex immunostaining in the dHC and cortical areas confirmed the results of confocal double immunostaining experiments, showing the highest levels of IGF-2R in excitatory and inhibitory neurons, followed by mural cells and hippocampal oligodendrocyte lineage cells, with low levels in vascular endothelial cells, astrocytes, microglia, and perivascular fibroblasts. In oligodendrocyte lineage cells, IGF-2R levels were higher in the dHC than in the CC but could not be determined in cortical regions.

### 3.8. IGF-2R Is Abundant in Cell Subpopulations in the Choroid Plexus and Meninges

**Figure 8.**
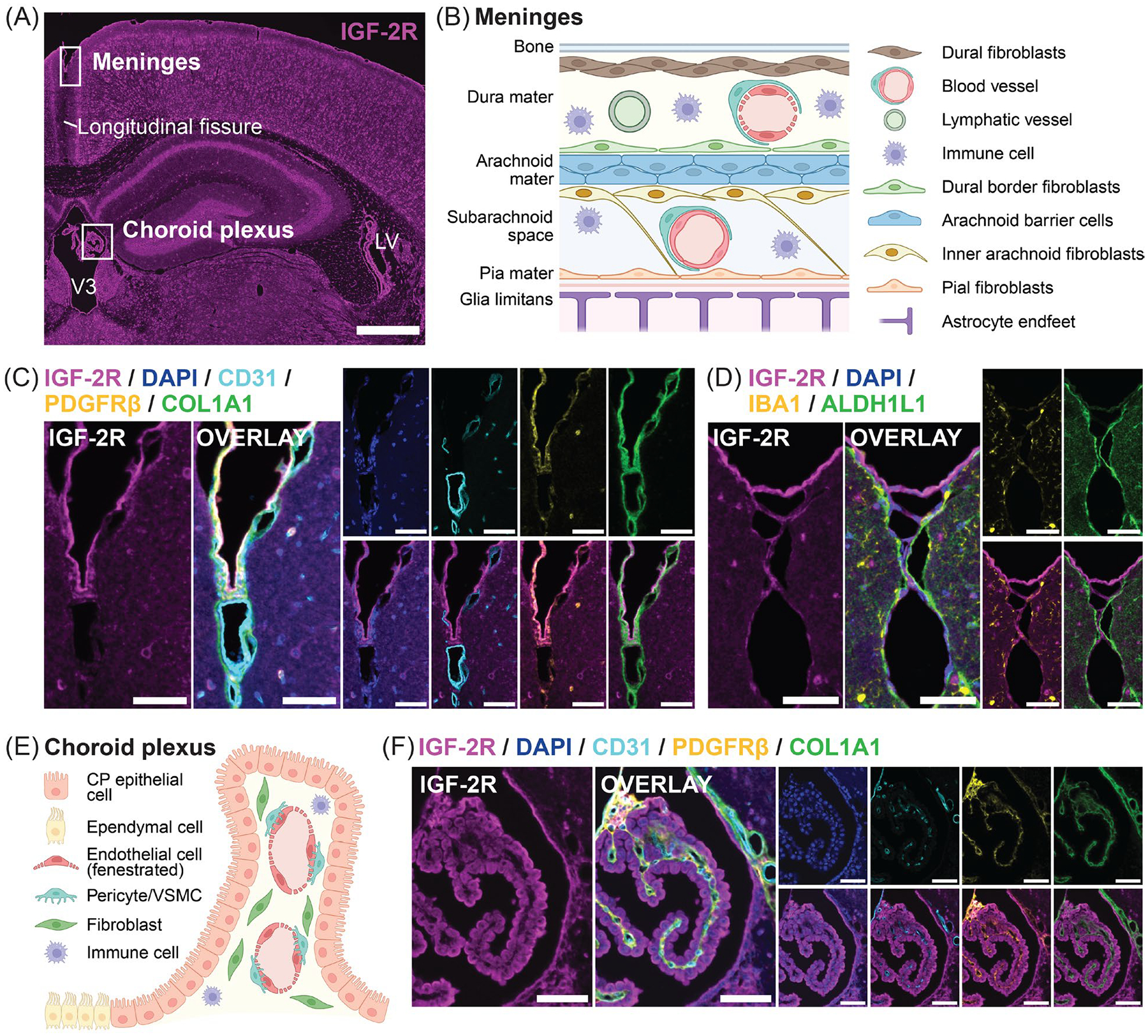
High levels of IGF-2R were found in cell populations in the ventricles and meninges. (**A**) ROIs in meninges and choroid plexus indicated by white boxes on a 20× fluorescent photomicrograph of IGF-2R signal (magenta) from multiplex immunostaining (scale bar = 500 μm). Locations of the lateral and third ventricles (LV and V3, respectively) are also indicated. (**B**) Schematic of meninges. Created in BioRender https://BioRender.com/g31ixv2 (accessed on 17 March 2025). (**C**) 20× fluorescent photomicrographs of IGF-2R, markers of vascular cell types, and DAPI in the meninges showing colocalization of IGF-2R with PDGFRβ. Images show single channels, overlays with IGF-2R, and an overlay of all channels. Channel is indicated by text color. Scale bars = 50 μm. (**D**) As (**C**) for IGF-2R and markers of astrocytes and microglia in the meninges. (**E**) Schematic of the choroid plexus. Created in BioRender https://BioRender.com/fqjx1hj (accessed on 17 March 2025). (**F**) As (**C**) for choroid plexus, showing that the distribution of IGF-2R is consistent with high levels in choroid plexus (CP) epithelial cells and ependymal cells lining the ventricle. VSMC—vascular smooth muscle cell.

We next assessed the distribution of IGF-2R and colocalization with markers of vasculature at the meninges, choroid plexus, and ventricles in images of multiplex TSA stainings (Figure 8A).

The meninges are composed of multiple layers of fibroblasts and fibroblast-like cells, vasculature, and immune cells and are located external to the glia limitans, a barrier formed by astrocyte endfeet and basement membrane (Figure 8B). IGF-2R, PDGFRβ, COL1A1, and ALDH1L1 each exhibited a strong, continuous narrow band of staining at or near the brain surface (Figure 8C,D). IGF-2R staining predominantly colocalized with PDGFRβ staining and was external to both ALDH1L1 (glia limitans) and intense COL1A1 staining. Moreover, IGF-2R and PDGFRβ were not detected along the longitudinal fissure, whereas strong COL1A1 staining was observed (Figure 8C). This distribution indicates that meningeal cells with high levels of IGF-2R are likely located in the arachnoid mater [73] (Figure 8B), where fibroblast-like cells expressing *Pdgfrb* and *Igf2r*, as well as substantially lower levels of *Col1a1* than pial fibroblasts, have been identified by single-cell RNA-Seq [74]. In the choroid plexus, which consists of a vascularized central stroma surrounded by specialized epithelial cells (Figure 8E), high intensity IGF-2R staining was observed that did not colocalize with vascular markers but, instead, matched the distribution of choroid plexus epithelial cells (Figure 8F). Relatively low intensity IGF-2R staining in the stroma colocalized mainly with PDGFRβ and COL1A1, but not with CD31 staining. Furthermore, high levels of IGF-2R were found in cells lining the lateral and third ventricles that did not express any of the cell-type-specific markers, consistent with ependymal cells (Figure 8F).

In summary, additional cell populations with high IGF-2R levels were identified based on distribution: choroid plexus epithelial cells, ependymal cells lining ventricles, and a subpopulation of meningeal cells.

### 3.9. Analysis of Igf2r Expression in Single-Cell RNA-Seq Databases

Finally, we analyzed *Igf2r* mRNA expression across mouse brain cell types in publicly available sequencing databases. Three brain-wide datasets were used: (1) single-cell RNA-Seq of the whole brain of juvenile male and female mice (PN12–30) [33]; (2) single-nucleus RNA-Seq of the whole brain of adult (PN56) male and female mice [34], and (3) single-cell RNA-Seq of 9 regions of adult (PN60–70) male mouse brain [35]. To dissect *Igf2r* expression in vascular cell subpopulations, we also analyzed data from a single-cell RNA-Seq study that isolated vascular fragments from the whole brains of 10-to 19-week-old transgenic reporter mice of both sexes [36]. Further details of these datasets can be found in Methods.

**Figure 9.**
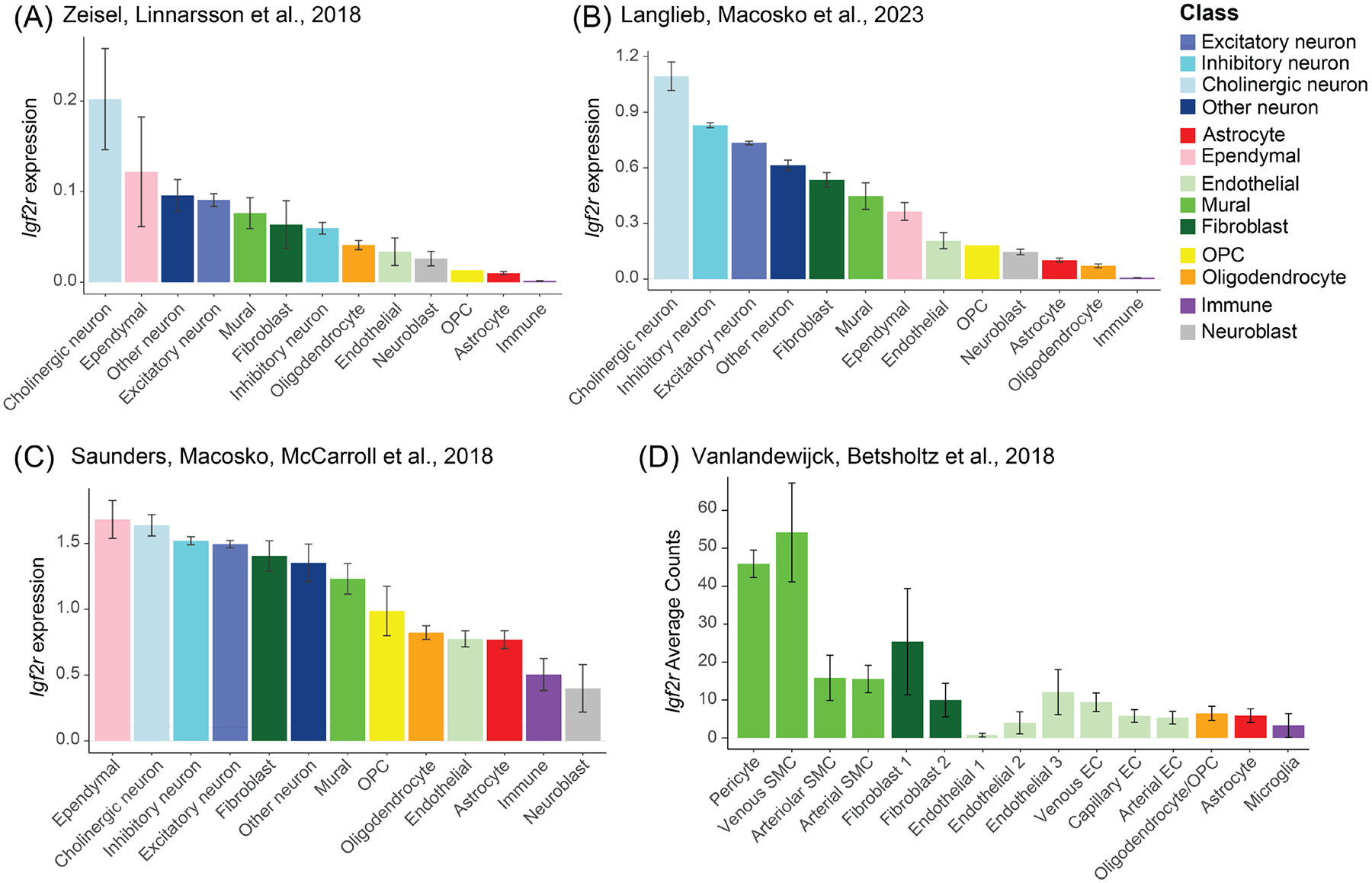
Expression of *Igf2r* mRNA in brain single-cell RNA sequencing databases. (**A**–**C**) Expression of *Igf2r* across cell classes in mouse brain in single-cell/nucleus RNA-Seq databases. Bar plots show mean ± SEM of aggregate expression values for cell populations in each class. (**A**) Single-cell RNA-Seq of juvenile mouse brain [33], (**B**) Single-nucleus RNA-Seq of adult mouse brain [34], (**C**) Single-cell RNA-Seq of nine regions of adult mouse brain [35]. (**D**) Average *Igf2r* counts for cell populations in single-cell RNA-Seq data isolated from adult mouse transgenic reporter lines targeting brain vascular cells [36].

The data revealed expression of *Igf2r* in most cell types, but at varying levels (Figure 9). In the dataset of Zeisel et al. [33] (Figure 9A), the highest *Igf2r* levels were observed in cholinergic neurons, with the highest expressing subpopulation located in the afferent nuclei of cranial nerves, in accordance with our finding that IGF-2R is highly enriched in the facial motor nucleus. Among non-neuronal cell populations, *Igf2r* was most highly enriched in choroid plexus epithelial cells (*Ttr*+). Among other brain ependymal populations, *Igf2r* expression was also enriched in the most abundant subtype (*Ccdc153*+, *Krt15*+), but low in subpopulations in the midbrain (*Tnnt3*+), spinal cord, and in hypendymal cells of the subcommissural organ. Among vascular cell populations, *Igf2r* was detected in all subtypes, with the highest levels in a pericyte subpopulation and arachnoid barrier cells (*Slc47a1*+) and the lowest levels in endothelial cells and a subtype of CNS fibroblasts expressing perivascular markers (*Pdgfra*+ *Lum*+ *Il33*+ *Ptgds*-). However, some of the subpopulations in this dataset had mixed expression of endothelial and pericyte markers, indicating incomplete separation of these cell types. Among oligodendrocyte lineage subpopulations, *Igf2r* levels were relatively similar, but lowest in OPCs. *Igf2r* levels were very low in astrocytes and almost undetectable in microglia.

In the Langlieb et al. dataset [34], cholinergic neuron populations again had the highest average level of *Igf2r*, followed by other neuronal populations, then fibroblasts, mural cells and ependymal cells (Figure 9B). Little to no *Igf2r* was detected in microglia, and levels were very low in astrocytes and oligodendrocytes, but slightly higher in OPCs and endothelial cells. Among mural cell subtypes identified by marker genes, *Igf2r* expression was highest in a subtype of contractile VSMCs (*Actg2*+ *Cnn1*+), followed by pericytes (high *Kcnj8*+), then other arterial/arteriolar VSMCs (*Acta2*+ *Olfr558*+), and lowest in putative venous VSMCs (low *Kcnj8*+ *Acta2*+ *Ccl19*+). Among the 37 fibroblast clusters identified in this study, *Igf2r* levels were variable among perivascular and meningeal fibroblasts and relatively high in the three stromal-like clusters, with the highest levels found in *Rbp4*+ perivascular subtypes (*Pdgfra*+ *Lum*+ *Dcn*+ *Eya2*+), *Slc47a1*+ meningeal subtypes (*Eya2*+ *Mgp*+ *Slc4a4*+), and stromal-like fibroblasts (*Vdr*+ *Col1a1*+ *Mmp13*+ *Bglap*+ *Pdgfra*+).

In the Saunders et al. dataset [35], a similar expression distribution was observed, with *Igf2r* levels highest in ependymal cells and cholinergic neurons, followed by other neuronal populations, fibroblasts, and mural cells, and lowest in immune cells and neuroblasts (Figure 9C). Within the hippocampus, *Igf2r* expression was highest in epithelial and ependymal cells, followed by neurons and mural cells, and was significantly higher in OPCs and committed oligodendrocyte precursors (*Tnr*+) compared to oligodendrocytes (*Trf*+; *p* = 0.00333), astrocytes (*p* = 0.00245), microglia (*p* = 0.00535) and endothelial cells (*p* = 0.00716). Across all cell clusters, *Igf2r* expression was highest in subpopulations of mural cells, deep-layer cortical pyramidal cells, ventral hippocampus neurons, fibroblasts, dividing cells of the oligodendrocyte lineage, and parvalbumin interneurons.

The vascular-enriched dataset of Vanlandewijck et al. [36] showed high expression of *Igf2r* in pericytes and venous VSMCs relative to arterial/arteriolar VSMCs, endothelial cells and glial populations. *Igf2r* was not enriched in fibroblasts relative to endothelial cells and glia, contrary to the other databases analyzed. This discrepancy compared to whole-brain datasets may be due to the isolation of only a specific subset of fibroblasts due to the choice of transgenic reporter line (Pdgfra-H2BGFP), as well as the small number of these cells (N = 86).

We also compared *Igf2r* expression with that of the gene coding for CD-M6PR, *M6pr*. Expression of *M6pr* was less variable among cell types, and the ranking of cell types by expression level was less consistent across datasets than for *Igf2r* (Figure S7). Notably, while *Igf2r* was enriched in mural cells compared to endothelial cells in all four datasets, *M6pr* had similar expression levels in the two cell types in the three brain-wide datasets, and lower relative enrichment in pericytes and VSMCs in the vascular cell-enriched dataset (Figure S7). In microglia, *M6pr* expression levels were similar to those in other cell types, whereas *Igf2r* levels were consistently very low to undetectable. Also, oligodendrocytes had relatively higher expression of *M6pr* than *Igf2r* compared to other cell types (i.e., oligodendrocytes were higher ranked in all the datasets for *M6pr* expression than for *Igf2r*). Whereas expression of *Igf2r* was substantially higher in ependymal cells than in oligodendrocytes in all datasets, *M6pr* had similar expression levels in these cell types in all datasets. Both receptors had low expression in neuroblasts compared to other cell types.

Collectively, these results confirmed that *Igf2r* mRNA is enriched in neurons, ependymal cells, mural cells and fibroblasts relative to glial populations, endothelial cells and neuroblasts.

## 4. Discussion

This study showed that IGF-2R protein is distributed throughout the mouse brain. Across the dHC, ACC, mPFC, and RSC—chosen for our analyses because of their critical roles in memory—the highest levels of IGF-2R were found in the somas of excitatory, inhibitory, and cholinergic neurons. The next highest level of IGF-2R enrichment was found in ependymal, meningeal, vascular mural cell, and oligodendrocyte lineage populations. Very low to undetectable levels were observed in astrocytes, microglia, vascular endothelial cells, and perivascular fibroblasts. Our mRNA analyses of previously published transcriptomic data confirmed that *Igf2r* expression is highest in neurons, particularly in cholinergic populations, and in mural cells relative to vascular endothelial cells [33–36]. *Igf2r* expression was also confirmed to be relatively high in ependymal cells, low in astrocytes and endothelial cells, and almost undetected in microglia. Additionally, these transcriptomic analyses revealed that *Igf2r* expression is low in neuroblasts.

Our immunostaining and mRNA data analyses are consistent with and significantly extend previous studies of the IGF-2R distribution in rodent brain [2,6,23,25,26,75]. Compared to the detailed rat brain-wide study from Hawkes and Kar [25], who used enzyme-linked immunohistochemistry, the main differences we observed were lower levels of receptor staining in the caudoputamen, lateral olfactory tract, and cortical layers IV and VI, positive staining in SLM interneurons, and similar (rather than lower) levels of staining in CA1 compared to CA3 pyramidal neurons. These differences are in agreement with another study in the mouse brain [6].

The analysis of IGF-2R expression in subcellular compartments revealed that IGF-2R is primarily concentrated in the somas, particularly in perinuclear regions, consistent with the known enrichment of IGF-2R in the Golgi apparatus and TGN [5]. The high expression of IGF-2R in neurons may relate to the high requirement for vesicle-dependent functions. Therefore, the level of IGF-2R expression may correlate with the size of neurons and the extension of their processes. In dendritic compartments identified by anti-MAP2 staining and in putative axonal compartments identified based on anatomical organization, a significant IGF-2R immunostaining with punctate organization was detected. The SLu and hilus showed distinctive IGF-2R staining patterns, as IGF-2R immunoreactivity not only colocalized with MAP2, but was also observed in the surrounding region, consistent with the conclusion that IGF-2R is localized in mossy fibers or their giant synaptic terminals. In support of the latter, colocalization of IGF-2R with the presynaptic scaffolding protein Bassoon has been reported in cultured hippocampal neurons [20]. Mossy fiber terminals have many unique properties, including their large size, complex shape, and exceptionally large pool of synaptic vesicles [76–78]. This large vesicle pool is accompanied by rapid clathrin-independent endocytosis, which may be required to support high firing rates [79–81]. From these data, we speculate that IGF-2R may be involved in the genesis or trafficking of pre- and postsynaptic vesicle pools. To our knowledge, there are currently no data available that support the hypothesis that IGF-2R plays a role in pre- or post-synaptic vesicle functions, and studies are needed to address this important question. Changes in the size of the readily releasable pool of vesicles are one of the mechanisms underlying the exceptionally strong presynaptic plasticity of mossy fiber synapses [82], supporting the idea that IGF-2R at mossy fiber terminals could play a direct role in vesicle trafficking or an indirect support role via protein degradation. Involvement of IGF-2R in vesicle formation could also explain its high level in neurons—cells that highly rely on vesicle physiology.

IGF-2R punctate staining in dendrites was observed in multiple brain regions, with the greatest concentration in the proximal apical dendrites of hippocampal and cortical pyramidal neurons. In DG SM and CA3 SO, IGF-2R levels were high compared to CA1 SO and SR, particularly distal from the soma, suggesting that these dendritic populations may have differential requirements for IGF-2R-mediated functions, such as increased levels of autophagy. These differences may be linked to the distinct electrophysiological properties of these dendritic populations, and future studies are needed to address these mechanistic questions. These intracellular distributions of IGF-2R are consistent with localization in multivesicular endosomes or endosomes, through which IGF-2R is known to be trafficked [83,84], and suggest that IGF-2R, in addition to its typical role in TGN trafficking of proteins such as lysosomal hydrolases, also plays roles in proximal and distal neuronal compartments. Because degradative lysosomes and lysosome-like organelles have been found in axons and dendrites as well as in somas [85], we speculate that the relatively high proportion of IGF-2R in axonal and dendritic compartments may reflect a role for IGF-2R in controlling the trafficking and degradation of locally synthesized proteins. This local protein metabolism regulation is critical for cognitive processes [86–88] and may underlie the key role of IGF-2R in memory consolidation [2,9]. Moreover, axonal endolysosomal trafficking, distribution and lysosomal functionality support neuronal health and homeostasis but become disrupted in aging and more significantly in several neurodegenerative diseases, contributing to protein aggregation—such as in Alzheimer’s and Parkinson’s diseases [85,89,90]. Thus, activating IGF-2R may represent a novel mechanism for developing new treatments for these conditions and diseases [14]. Future research should explore the therapeutic effects and underlying mechanisms of IGF-2R ligand treatments in these diseases, in addition to neurodevelopmental disorders and aging. Their positive outcomes could be transformative for many devastating diseases awaiting treatments.

A comparison of the mouse brain-wide distribution of relative protein levels with CD-M6PR revealed that, although both receptors are distributed throughout the brain, the regions with a greater enrichment of IGF-2R primarily contain projection neurons (i.e., mitral layer of olfactory bulb, layer II of olfactory tubercle, ventral and lateral groups of thalamic nuclei, and subiculum). Lower enrichment of IGF-2R was found in the hypothalamus, anterior and reticular groups of thalamic nuclei, and midbrain, including the substantia nigra pars compacta (SNpc). This distribution suggests differential roles of the two M6P receptors in the brain, which currently remain unknown. The differential distributions may indicate that IGF-2R plays a distinct role in neuronal populations with fast neurotransmission and long-range projections, whereas the CD-M6PR may be more engaged in neuronal populations involved in modulatory functions, with slower signaling by neuromodulators and neuropeptides (e.g., orexin, vasopressin, and oxytocin neurons in the hypothalamus, and dopamine in SNpc). Further studies are needed to investigate whether CD-M6PR and IGF-2R play specialized roles in regions critical for memory and cognition. Further experiments comparing the colocalization of the two receptors with markers of additional neuronal populations, such as aminergic systems, are needed to explore this hypothesis. Relatively low levels of IGF-2R in putative dopaminergic neurons in the SNpc may also have therapeutic implications, as this is the main population affected in Parkinson’s Disease, and IGF-2 has been shown to be neuroprotective and reduce α-synuclein accumulation in these cells [91,92]. IGF-2R was also notably enriched in mitral and granule cells of the olfactory bulb—populations that are known to undergo significant neurodegeneration in aging, neurological and neuropsychiatric disorders, positioning them as potential disease markers [93,94]. Another feature of these populations is that they communicate via dendro-dendritic synapses [95], suggesting the hypothesis that IGF-2R contributes to this mechanism. Furthermore, IGF-2R, but not CD-M6PR, exhibited diffuse staining in most regions, particularly in cortical layer I, the hilus and SLu of the hippocampus, caudoputamen, and thalamus, suggesting that IGF-2R is relatively abundant in neuronal processes, whereas CD-M6PR is more confined to the soma.

The relatively high presence of IGF2R in vascular mural cells, particularly in pericytes, suggests its role in vascular functions. Pericytes are mural cells found at intervals along the walls of capillaries and post-capillary venules. In the CNS, they play a key role in blood vessel formation, maintenance of the blood–brain barrier (BBB), regulation of immune cell entry to the central nervous system, and control of brain blood flow [96]. As IGF-2R has been shown to be expressed in the BBB and to transport IGF-2 across this structure [97], according to the findings, it is reasonable to believe that pericytes are important for this transport. A high level of IGF-2 has also been found in pericytes relative to several other cell types in the hippocampus, where it plays a crucial role in memory formation [98]. The dual expression of ligand and IGF-2R in pericytes suggests a possible autocrine role of IGF-2 in these cells, in addition to a pericyte-neuronal cooperation necessary for long-term memory [98–100]. Pericytes are also important for protein degradation, via the lysosomal pathway, in the vascular structure, including degradation of proteins within the BBB [101]. They degrade proteins such as α-synuclein and β-amyloid [102,103], in line with their major implication in neurodegenerative diseases such as Parkinson’s and Alzheimer’s diseases [104]. Another important role of IGF-2R, characterized in the peripheral vascular system and in cell cultures, is the regulation of angiogenesis, including the recruitment of endothelial progenitor cells [105,106]. Despite these findings, vascular endothelial cells had relatively low expression of IGF-2R protein and mRNA. This expression, however, may be developmentally regulated, since significant expression of IGF-2R in endothelial cells has been reported in the brains of neonatal mice up to 2–3 weeks of age and adolescent humans and primates [24]. Such developmental expression may facilitate M6P-dependent transport of lysosomal enzymes across the BBB observed in neonatal and adolescent but not adult mice [107,108].

The enrichment of IGF-2R protein and mRNA in cell populations associated with barriers between the brain, blood, and/or cerebrospinal fluid (CSF), i.e., choroid plexus epithelial cells, ependymal cells, and a meningeal fibroblast-like population likely located in the arachnoid mater, suggests its possible role in the regulation of CSF production and protein clearance. Future studies are needed to investigate this important question. Among fibroblasts, *Igf2r* mRNA levels analyzed in public databases were found relatively high on average, but with variable expression across subpopulations. Notably, differing gene expression profiles were detected both between and within the three main groups of brain fibroblasts: perivascular, stromal/choroid plexus, and meningeal [34,73]. Our immunostaining data showed high IGF-2R levels in a subset of meningeal cells, consistent with high mRNA expression in *Slc47a1*+ meningeal fibroblasts in two of the RNA databases [33,34]. Subpopulations of perivascular fibroblasts with very low *Igf2r* levels were also found in both datasets, in line with our protein-level data in COL1A1+ cells in the hippocampus and cortex. It should be emphasized here that research into the molecular and functional diversity of brain fibroblast populations has been very limited to date.

IGF-2R enrichment in a subset of oligodendrocyte lineage cells was evident in the dHC as well as in the CC, but could not be determined in cortical regions due to overlap with neuron somas. IGF-2R has previously been observed in OLIG2-positive cells in the CC and cortex of neonatal rats in a white matter injury model [109], but a more comprehensive survey of its expression in healthy brains, to our knowledge, has not yet been performed. Our analysis of single-cell RNA-Seq databases found that *Igf2r* expression in oligodendrocyte lineage populations was generally low, at similar levels to astrocytes and endothelial cells. However, in the region-specific dataset by Saunders et al. [35], *Igf2r* in the hippocampus was enriched in oligodendrocyte precursors compared to oligodendrocytes and other glia, which may correspond to the IGF-2R enrichment we observed in a subset of hippocampal oligodendrocytes.

In astrocytes and microglia, IGF-2R immunostaining could not be conclusively distinguished from background, indicating that IGF-2R is either absent from these cells or present at very low levels. In both the RNA databases and immunostainings of regions with minimal diffuse staining, levels of IGF-2R in astrocytes were higher than in microglia, suggesting that low levels of IGF-2R are found in astrocytes. Although differential levels of IGF-2R in various cell types may be linked to their distinct functions and intracellular structures, one could speculate that the absence of IGF-2R in astrocytes and microglia may also contribute to the early stages of neurodegenerative diseases [110]. In addition, our analyses of IGF-2R protein levels in glial and vascular cell types mainly focused on the hippocampus and cortex; IGF-2R levels in these cell types may differ across brain regions, as observed for neurons.

A limitation of the present study is the use of only male mice for immunostaining experiments; the IGF-2R distribution in females shall be addressed in future studies. To our knowledge, no studies have reported sex differences in IGF-2R levels. Another limitation of this investigation is the reliance on a single antibody to IGF-2R. This antibody, however, presents the advantages of being monoclonal and having been validated by knockout in brain sections [2,111]. Further studies should investigate IGF-2R expression in human tissues, as differences may exist compared to rodents. Understanding IGF-2R distribution in the human brain is essential for translational applications and a better understanding of its links to human neurological diseases.

Future work should expand on the characterization of IGF-2R protein levels in mural, fibroblast, and oligodendrocyte subpopulations, as well as in all types of neurons, including aminergic populations. Additional investigations of the subcellular compartment localization are also needed, for example, using electron microscopy or subcellular fractionation. Differences in IGF-2R levels and cellular distribution over development, aging, and under pathological conditions should also be addressed, and are of particular importance for translational research. A critical outstanding question is the nature of all mechanisms and targets controlled by IGF-2R and how its roles may differ across cell types and subcellular compartments. Finally, as the members of the insulin/IGF system—namely, IGF-1, IGF-2, insulin, and their respective high-affinity receptors—are known to cooperate and antagonize one another, the function and regulation of IGF-2R in brain cell types should also be addressed in relation to the other members of the system. These interactions are likely to be critical for brain development, mature functioning, aging, and disease.

In conclusion, IGF-2R is highly expressed throughout the adult brain, with very different levels in various brain regions and cell types. The high IGF-2R expression in neurons, including neuronal processes, suggests its contribution to vesicle-mediated protein metabolism or quality control functions, which may serve distinct roles in the soma vs. processes. The high levels of IGF-2R in mural cells and ependymal cells may be related to contributions to protein degradation at barriers between the blood, brain and CSF. Our data suggest that IGF-2R is involved differentially in different circuits and cell-type functions.

## Supplementary Material

Supplementary figures and tables

**Supplementary Materials:** The following supporting information can be downloaded at https://www.mdpi.com/article/10.3390/receptors5010001/s1. Figure S1: Quantification methods for multiplex tyramide signal amplification data; Figure S2: Distribution of IGF-2R in brain subregions; Figure S3: Single-channel images for double staining of IGF-2R and neuronal markers; Figure S4: Double staining of IGF-2R and an alternative astrocyte marker (ALDH1L1); Figure S5: Extended analysis of multiplex tyramide signal amplification data; Figure S6: IGF-2R levels in CaMK2α+ and GAD67+ cells in cortical regions in multiplex immunostaining; Figure S7: Expression of cation-dependent M6pr mRNA in brain single-cell RNA sequencing databases; Table S1: Differences in IGF-2R levels between brain cell types (supporting Figure 7D); Table S2: Differences in IGF-2R levels between cell types in hippocampal subregions (supporting Figure S5C,D); Table S3: Differences in IGF-2R levels between glial populations in the dorsal hippocampus and corpus callosum (supporting Figure S5E).

## Figures and Tables

**Table 1.** Antibodies used to target specific cell types and subcellular compartments in immunofluorescent double staining experiments.

Target Protein	Cell Type	Subcellular Localization	Cat. #, Vendor	Species	Dilution Factor
CaMK2α	Excitatory neurons	Soma and processes	05–532, Sigma-Aldrich, St. Louis, MO, USA	Mouse	300
GAD67	Inhibitory neurons	Soma and processes	MAB5406, Sigma-Aldrich, St. Louis, MO, USA	Mouse	2000
ChAT	Cholinergic neurons	Soma and processes	AB144P, Sigma-Aldrich, St. Louis, MO, USA	Goat	500
MAP2	Neurons	Soma and dendrites	ab92434, Abcam, Cambridge, UK	Chicken	2000
PSD95	Neurons	Dendritic spines	MA1045, Invitrogen, Waltham, MA, USA	Mouse	1000
GFAP	Astrocytes	Soma and processes	AB5541, Sigma-Aldrich, St. Louis, MO, USA	Chicken	1000
ALDH1L1	Astrocytes	Soma and processes	ab56777, Abcam, Cambridge, UK	Mouse	500
IBA1	Microglia	Soma and processes	234308, Synaptic Systems, Göttingen, Germany	Guinea pig	500
IBA1	Microglia	Soma and processes	234009, Synaptic Systems, Göttingen, Germany	Chicken	750
SOX10	Oligodendrocyte lineage cells	Nucleus	AF2864, R&D systems, Minneapolis, MN, USA	Goat	200
CD31	Vascular endothelial cells	Cell membrane	AF3628, R&D systems, Minneapolis, MN, USA	Goat	150
PDGFRβ	Pericytes and vascular smooth muscle cells	Cell membrane	14-1402-82, Invitrogen, Waltham, MA, USA	Rat	100

**Table 2.** Antibodies and experimental conditions used for multiplex tyramide signal amplification experiments.

Panel	Target Protein	Clone	Cat #, Vendor	Species	Dilution Factor	Retrieval Protocol	Fluorophore and Dilution
1	CaMK2α	6G9	05–532, Sigma-Aldrich, St. Louis, MO, USA	Mouse	700	ER1–20	520 (1:150)
1	GAD67	1G10.2	MAB5406, Sigma-Aldrich, St. Louis, MO, USA	Mouse	500	ER2–20	620 (1:150)
1	PDGFRβ	28E1	3169, CST, Danvers, MA, USA	Rabbit	100	ER2–60	570 (1:150)
1	CD31	D8V9E	77699, CST, Danvers, MA, USA	Rabbit	300	ER2–20	480 (1:200)
1	COL1A1	E8F4L	72026, CST, Danvers, MA, USA	Rabbit	150	ER2–20	690 (1:150)
1	IGF-2R	EPR6599	ab124767, Abcam, Cambridge, UK	Rabbit	3000	ER2–20	780 (1:25)
2	IBA1	Poly	019–19471, Wako, Richmond, VA, USA	Rabbit	250	ER1–20	620 (1:150)
2	ALDH1L1	Poly	ab87117, Abcam, Cambridge, UK	Rabbit	5000	ER2–20	570 (1:150)
2	OLIG2	Poly	AB9610, Sigma-Aldrich, St. Louis, MO, USA	Rabbit	100	ER2–60	480 (1:200)
2	IGF-2R	EPR6599	ab124767, Abcam, Cambridge, UK	Rabbit	5000	ER2–20	690 (1:150)

## Data Availability

The raw data supporting the conclusions of this article will be made available by the authors on request.

## Abbreviations

The following abbreviations are used in this manuscript:

ACC: anterior cingulate cortex
ALDH1L1: aldehyde dehydrogenase family 1 member L1
ANN: artificial neural network
AU: arbitrary units
BBB: blood–brain barrier
CA1–3: cornu ammonis 1–3
CaMK2α: calcium/calmodulin-dependent protein kinase II subunit alpha
ChAT: choline acetyltransferase
CC: corpus callosum
CD31: cluster of differentiation 31
CD-M6PR: cation-dependent mannose-6-phosphate receptor
CNS: central nervous system
COL1A1: collagen type I alpha 1 chain
CSF: cerebrospinal fluid
DAPI: 4′6-diamidino-2-phenylindole
DG: dentate gyrus
dHC: dorsal hippocampus
GAD67: glutamate decarboxylase 67
GFAP: glial fibrillary acidic protein
HRP: horseradish peroxidase
IBA1: ionized calcium-binding adaptor molecule 1
IGF-2: insulin-like growth factor 2
IGF-2R: insulin-like growth factor 2 receptor
MAP2: microtubule-associated protein 2
M6P: mannose-6-phosphate
MOC: Mander’s overlap coefficient
mPFC: medial prefrontal cortex
OPC: oligodendrocyte precursor cell
PB: phosphate buffer
PBS: phosphate-buffered saline
PBST: phosphate-buffered saline with Triton X-100
PDGFRβ: platelet-derived growth factor receptor beta
PFA: paraformadehyde
PN: postnatal day
PSD95: postsynaptic density protein 95
ROI: region of interest
RSC: retrosplenial cortex
SEM: standard error of the mean
SG: stratum granulosum
SLu: stratum lucidum
SLM: stratum lacunosum moleculare
SM: stratum moleculare
SO: stratum oriens
SOX10: sex-determining region Y (SRY)-box transcription factor 10
SP: stratum pyramidale
SR: stratum radiatum
SNpc: substantia nigra pars compacta
TGN: trans-Golgi network
TSA: tyramide signal amplification
VSMC: vascular smooth muscle cell
