# Supplementary material for: Distribution and Levels of Insulin-like Growth Factor 2 Receptor Across Mouse Brain Cell Types
Source: Receptors (Basel). Author manuscript; Available in PMC 2026 Feb 24. (PMC12922683; doi:10.3390/receptors5010001)
Supplement: Supplementary figures and tables [file NIHMS2145035-supplement-Supplementary_figures_and_tables.pdf]

Supplementary information for: Distribution and levels of insulin-like growth factor 2 receptor across mouse brain cell types

Jessica R. Gaunt, Gokul Manoj and Cristina M. Alberini

Supplementary Figures

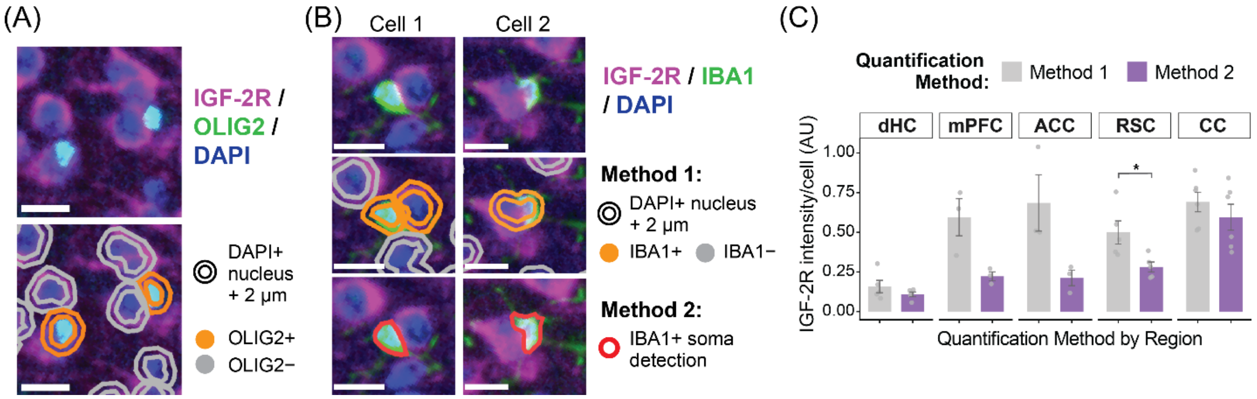

Figure S1: Quantification methods for multiplex tyramide signal amplification data

(A) Examples of satellite oligodendrocytes in the ACC, showing multiplex immunostaining of IGF-2R and OLIG2 with DAPI-stained nuclei (left), and annotation of DAPI+ nuclei detection and quantification area (discs; right). Classification of DAPI+ discs as OLIG2+/− is indicated by line color. Scale bars = 10 µm. (B) Examples of satellite microglia in the ACC, showing multiplex immunostaining of IGF-2R and IBA1 with DAPI-stained nuclei (top) and annotation of DAPI+ nuclei detection, discs, and classification as IBA1+/− (middle; Method 1). To reduce overlap with neurons, bright IBA1 staining in somas and nuclei was used to detect IBA1+ cell bodies and define the quantification area (bottom; Method 2). Scale bars = 10 µm. (C) Comparison of results of methods shown in (B) for quantification of mean IGF-2R intensity/cell in arbitrary units (AU) in each ROI. Significant results of t-tests comparing methods within each region are indicated (* $p < 0.05$). Data are presented as mean ± SEM and points correspond to individual subject means.

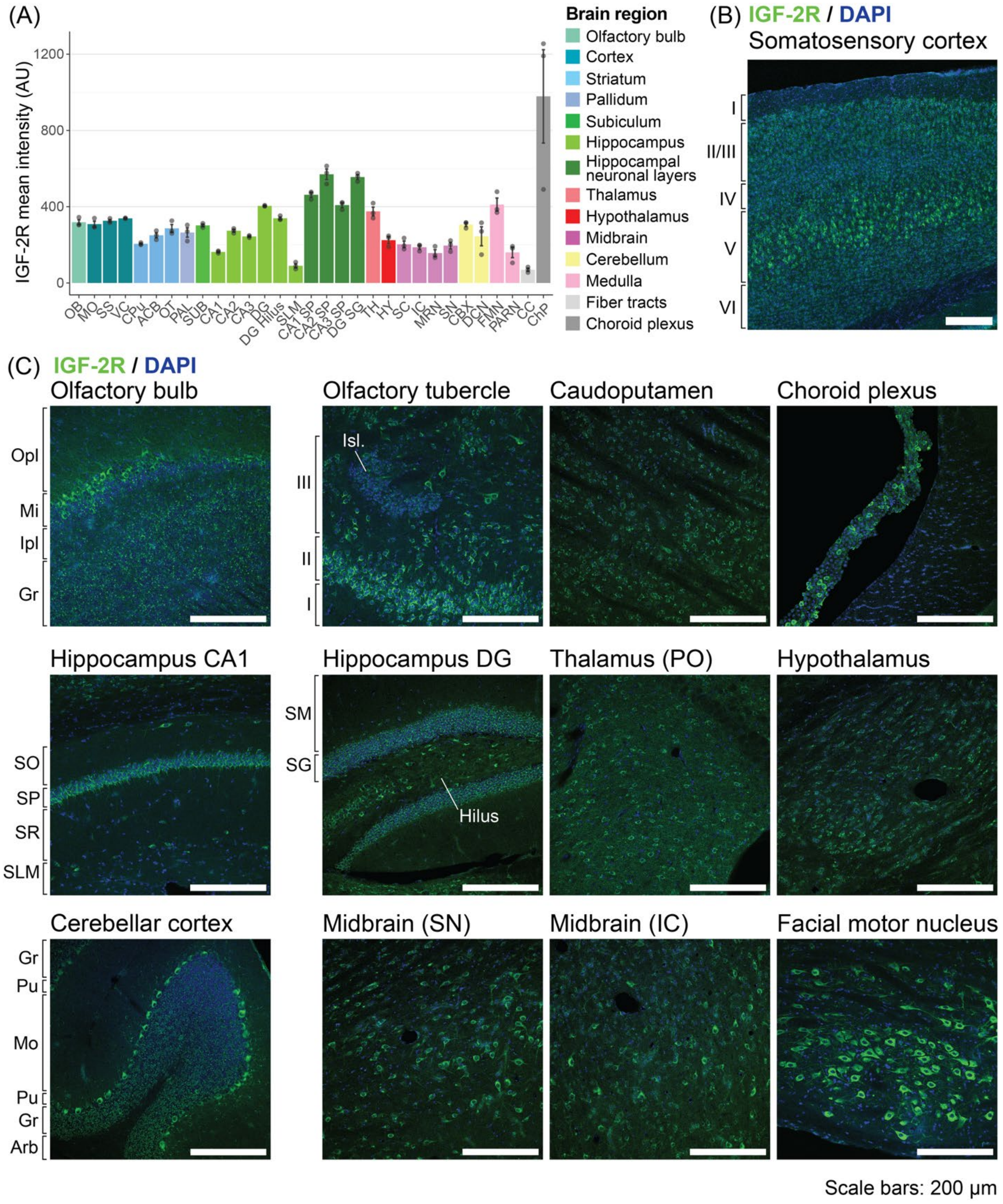

Figure S2: Distribution of IGF-2R in brain subregions.

(A) Mean IGF-2R staining intensity in each ROI in arbitrary units (AU; N=3 anti-IGF-2R, N=2 Control). Data are presented as mean ± SEM and points correspond to individual subject means. (B-C) Representative confocal photomicrographs of immunofluorescent IGF-2R and DAPI staining in a sagittal section of adult mouse brain. Scale bars 200 µm. (B) 10X image of somatosensory cortex with annotated cortical layers. (C) 20X images of different brain regions with annotated layers and features.

Abbreviations: OB—Olfactory Bulb; MO—Somatomotor Cortex; SS—Somatosensory Cortex; VC—Visual Cortex; CPu—Caudoputamen; ACB—Nucleus Accumbens; OT—Olfactory Tubercle; PAL—Pallidum; SUB—Subiculum; CA1–3—Cornu Ammonis 1–3; DG—Dentate Gyrus; SLM—Stratum Lacunosum Moleculare; SP—Stratum Pyramidale; SG—Stratum Granulosum; TH—Thalamus; HY—Hypothalamus; SC—Superior Colliculus; IC—Inferior Colliculus; MRN—Midbrain Reticular Nucleus; SN—Substantia Nigra; CBX—Cerebellar Cortex; DCN—Deep Cerebellar Nuclei; FMN—Facial Motor Nucleus; PARN—Parvicellular Reticular Nucleus; CC—Corpus Callosum; ChP—Choroid Plexus; Opl—Outer plexiform layer; Mi—Mitral layer; Ipl—Inner plexiform layer; Gr—Granule layer; Isl.—Island of Calleja; SO—Stratum oriens; SR—stratum radiatum; SM—stratum moleculare; PO—Posterior complex; Pu—Purkinje layer; Mo—Molecular layer; Arb—Arbor vitae.

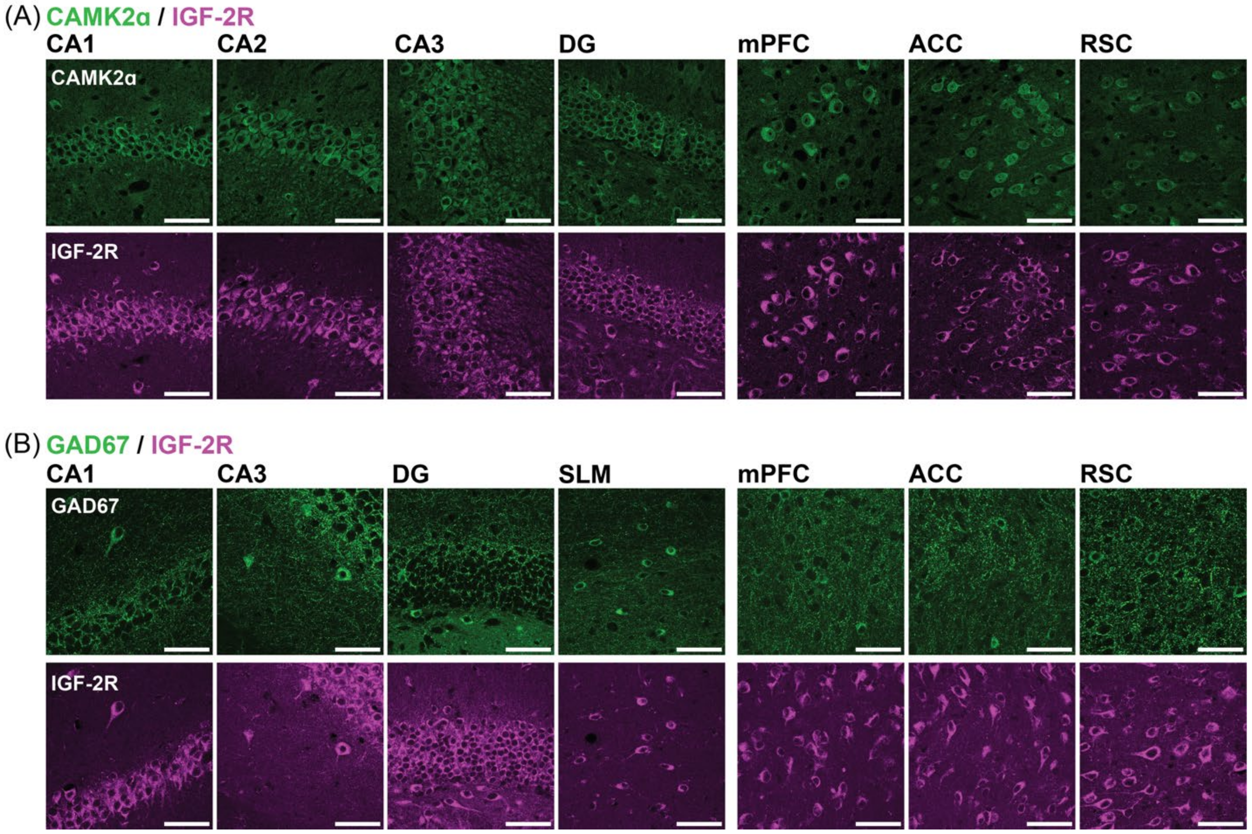

Figure S3: Single-channel images for double staining of IGF-2R and neuronal markers

Representative 63X confocal single-channel images of immunofluorescent double staining in dorsal hippocampus subregions (CA1-3, Dentate Gyrus [DG]), and cortical regions mPFC, ACC, and RSC. Scale bars 50 μm. (A) IGF-2R and CAMK2α. (B) IGF-2R and GAD67.

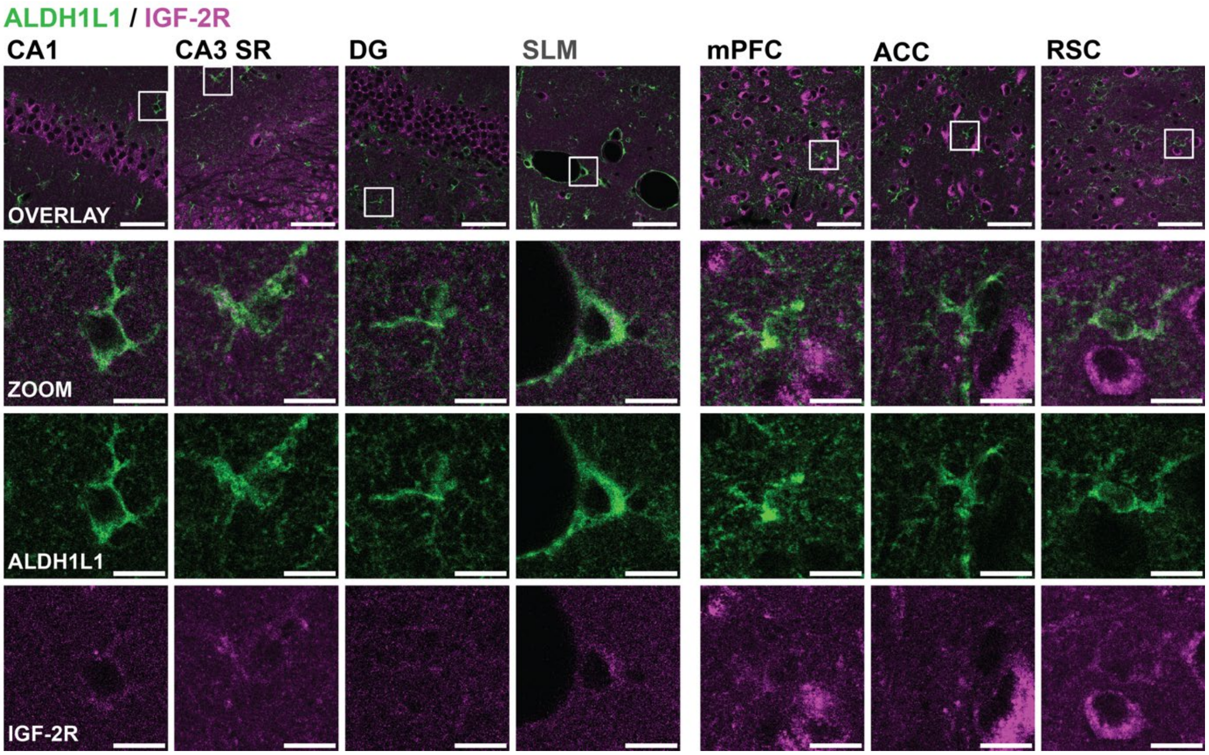

Figure S4: Double staining of IGF-2R and an alternative astrocyte marker (ALDH1L1). Representative 63X confocal image overlays of immunofluorescent double staining of IGF-2R and ALDH1L1 in dorsal hippocampus subregions (CA1-3, Dentate Gyrus [DG]), and cortical regions mPFC, ACC, and RSC (scale bars = 50 μm) with enlargements of selected cells showing single channel and overlay images (scale bars = 10 μm).

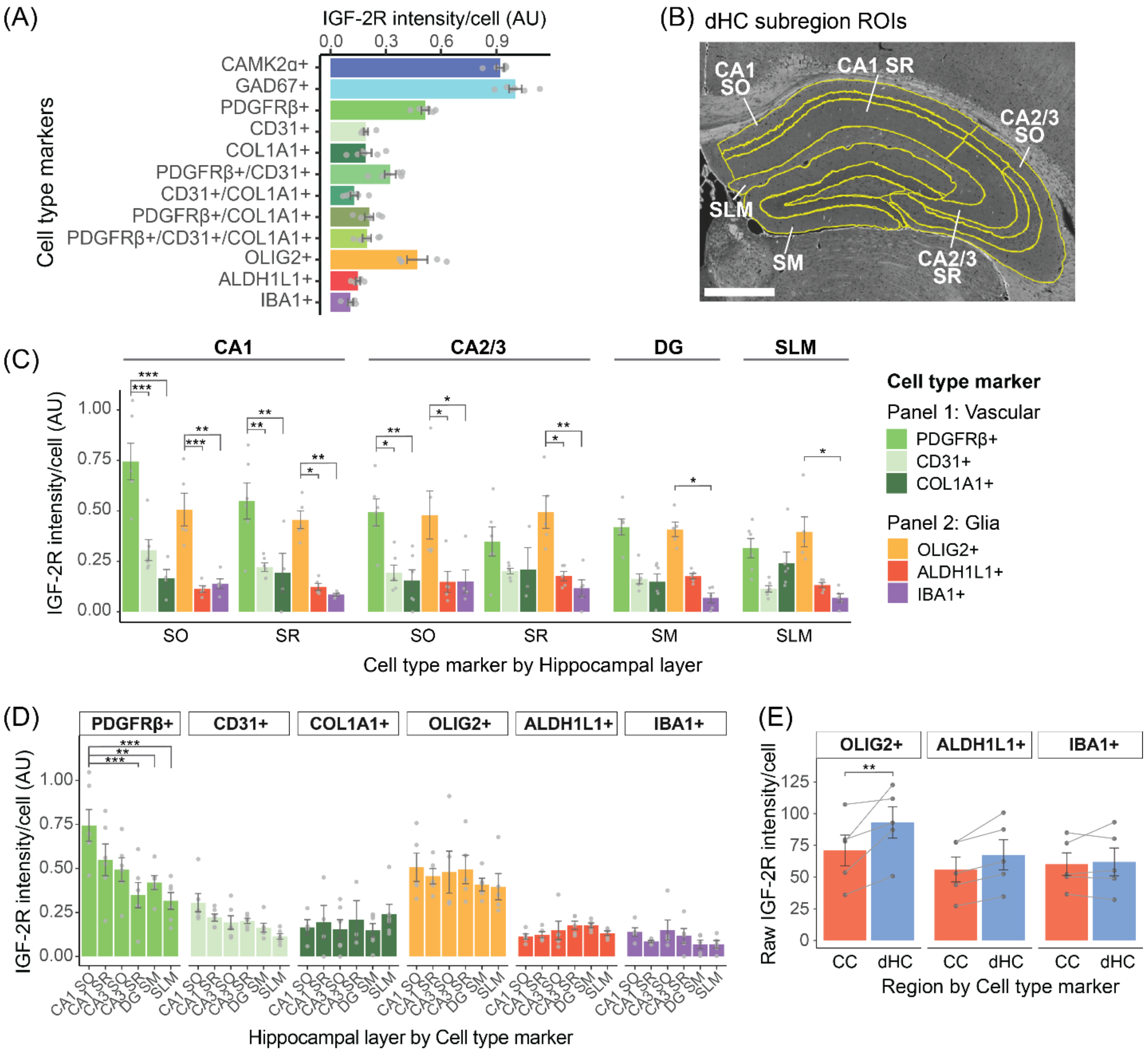

Figure S5: Extended analysis of multiplex tyramide signal amplification data

(A) Quantification of mean IGF-2R intensity/cell in arbitrary units (AU) in populations positive for each cell type marker and each combination of vascular markers in the dorsal hippocampus (dHC; N = 5–6). Data are presented as mean ± SEM and points correspond to individual subject means. (B) dHC subregion ROIs annotated on an image of tissue autofluorescence from Vectra Polaris scans (scale bar = 500 µm). (C) Mean normalized IGF-2R intensity/cell for glia and vascular markers in dHC subregions (N = 5-6 subjects). ROIs are grouped by hippocampal subfield

and layer. Differences in IGF-2R levels between markers and subregions were analyzed using a two-way ANOVA with Tukey's post hoc tests. Significant results of post hoc tests between markers in the same subregion and panel are indicated (* $p < 0.05$, ** $p < 0.01$, *** $p < 0.001$; Table S2). (D) As (C), with data grouped by cell type marker, indicating results of post hoc tests between subregions for each marker. (E) Raw mean IGF-2R intensity/cell in the dHC and CC for glial markers. Data are presented as mean ± SEM and lines indicate data points from the same image. Differences in IGF-2R levels between markers and regions were analyzed using a two-way ANOVA with subject as a blocking factor followed by Tukey's post hoc tests. Significant results of post hoc tests between regions are indicated for each cell type marker (Table S3).

Abbreviations: dHC—Dorsal Hippocampus; mPFC—Medial Prefrontal Cortex; ACC—Anterior Cingulate Cortex; RSC—Retrosplenial Cortex; CC—Corpus Callosum; CA1–3—Cornu Ammonis 1–3; DG—Dentate Gyrus; SO—Stratum oriens; SR—stratum radiatum; SM—stratum moleculare; SLM—Stratum Lacunosum Moleculare.

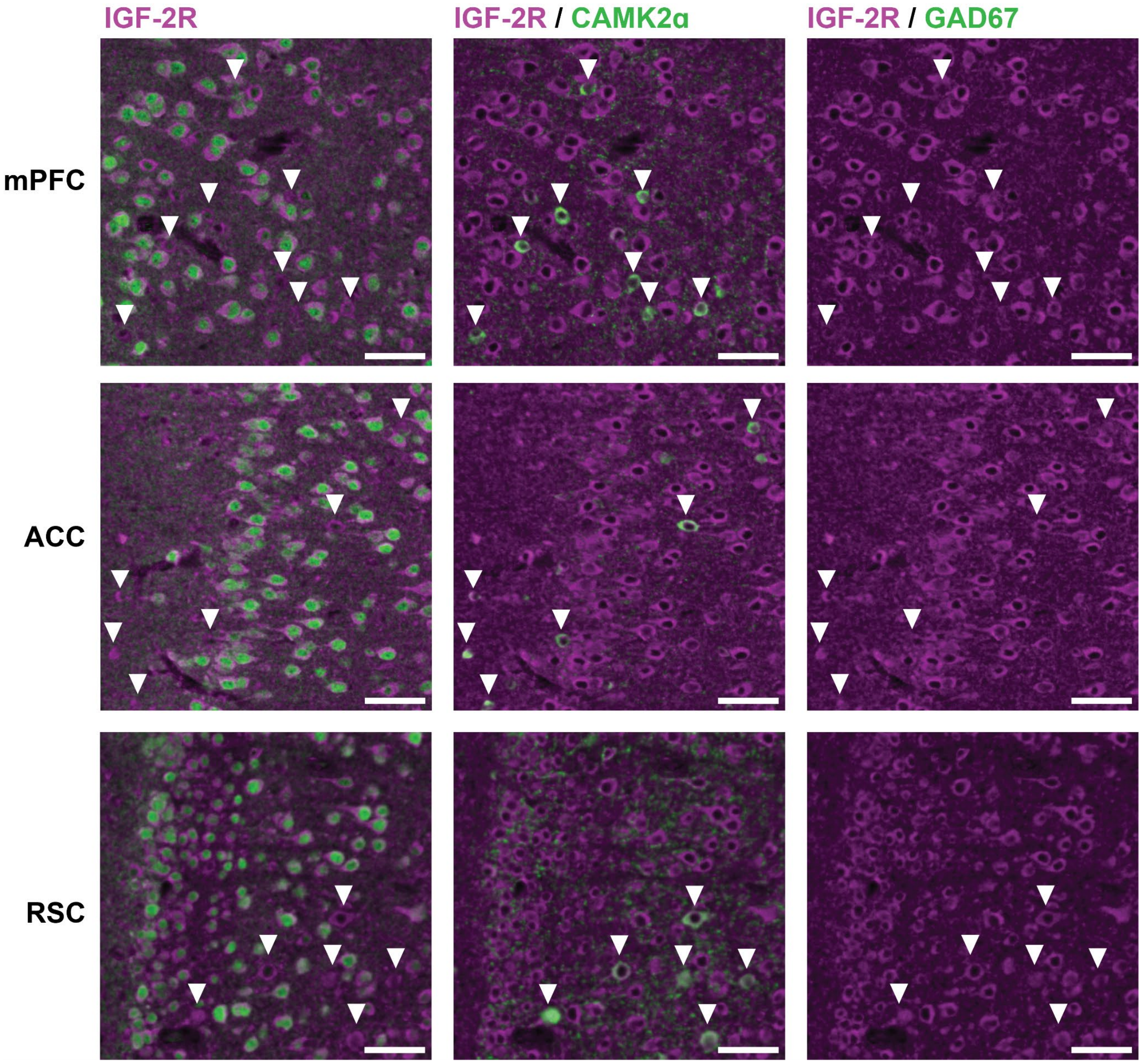

Figure S6: IGF-2R levels in CaMK2α+ and GAD67+ cells in cortical regions in multiplex immunostaining

Representative 20X fluorescent photomicrographs of multiple-label tyramide signal amplification (TSA) immunostaining for IGF-2R, and overlays of IGF-2R with CaMK2α, and GAD67 in the cortical regions mPFC, ACC and RSC. GAD67+ cells are indicated by white arrows in all images. IGF-2R levels in GAD67+ and CaMK2α+ somas were similar in the RSC, but lower in GAD67+ somas in the mPFC (Fig. 7). Scale bars = 50 μm.

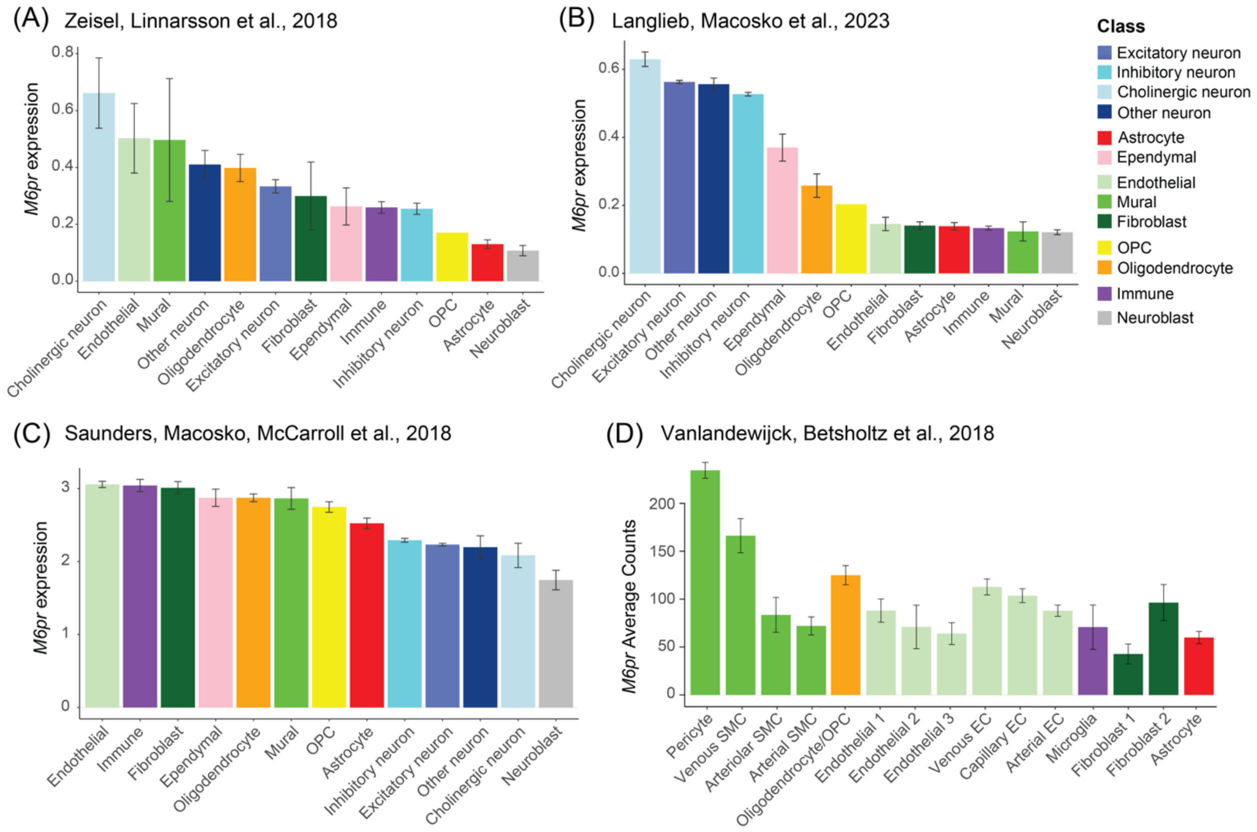

Figure S7: Expression of cation-dependent *M6pr* mRNA in brain single-cell RNA sequencing databases

(A-C) Expression of *M6pr* across cell classes in mouse brain in single-cell/nucleus RNA-Seq databases. Barplots show mean ± SEM of aggregate expression values for cell populations in each class. (A) Single-cell RNA-Seq of juvenile mouse brain (Zeisel et al., 2018). (B) Single-nucleus RNA-Seq of adult mouse brain (Langlieb et al., 2023). (C) Single-cell RNA-Seq of nine regions of adult mouse brain (Saunders et al., 2018). (D) Average *M6pr* counts for cell populations in single-cell RNA-Seq data isolated from adult mouse transgenic reporter lines targeting brain vascular cells (Vanlandewijck et al., 2018).

Supplementary Tables

Table S1 supporting Figure 7D: Differences in IGF-2R levels between brain cell types

Differences in normalized mean IGF-2R intensity between cell populations identified by cell-type-specific markers in multiplex immunostained sections, analyzed within each ROI (dHC, mPFC, ACC, RSC, CC) using one-way ANOVAs followed by Tukey's post hoc tests. Statistics presented for post hoc tests are mean difference (MD) between groups and p-value adjusted for multiple comparisons.

Contrast	dHC	mPFC	ACC	RSC	CC
Main effect of cell type	$F(7,37) = 162.400$, $p < 2E-16$	$F(7,16) = 8.233$, $p = 2.58E-4$	$F(7,16) = 11.990$, $p = 2.61E-5$	$F(7,32) = 85.450$, $p < 2E-16$	$F(6,35) = 13.610$, $p = 6.57E-8$
GAD67+-CAMK2α+	MD = 0.082, p = 4.07E-01	MD = -0.306, p = 3.10E-01	MD = -0.185, p = 6.93E-01	MD = -0.074, p = 8.06E-01	
PDGFRβ+-CAMK2α+	MD = -0.407, p = 2.42E-11	MD = -0.433, p = 5.80E-02	MD = -0.391, p = 4.11E-02	MD = -0.614, p = 2.87E-12	
CD31+-CAMK2α+	MD = -0.728, p = 0	MD = -0.671, p = 1.61E-03	MD = -0.648, p = 4.53E-04	MD = -0.825, p = 1.01E-13	
COL1A1+-CAMK2α+	MD = -0.729, p = 0	MD = -0.66, p = 1.92E-03	MD = -0.695, p = 2.10E-04	MD = -0.78, p = 1.05E-13	
OLIG2+-CAMK2α+	MD = -0.45, p = 5.37E-12	MD = -0.26, p = 4.94E-01	MD = -0.239, p = 4.12E-01	MD = -0.515, p = 2.61E-10	
ALDH1L1+-CAMK2α+	MD = -0.772, p = 0	MD = -0.735, p = 6.32E-04	MD = -0.677, p = 2.83E-04	MD = -0.768, p = 1.07E-13	
IBA1+-CAMK2α+	MD = -0.812, p = 0	MD = -0.652, p = 2.14E-03	MD = -0.638, p = 5.43E-04	MD = -0.724, p = 1.35E-13	
PDGFRβ+-GAD67+	MD = -0.49, p = 0	MD = -0.128, p = 9.68E-01	MD = -0.206, p = 5.84E-01	MD = -0.54, p = 7.81E-11	MD = -0.615, p = 1.55E-03

CD31+-GAD67+	MD = -0.811, p = 0	MD = -0.366, p = 1.49E-01	MD = -0.463, p = 1.16E-02	MD = -0.751, p = 1.13E-13	MD = -1.004, p = 3.80E-07
COL1A1+-GAD67+	MD = -0.812, p = 0	MD = -0.354, p = 1.73E-01	MD = -0.509, p = 5.09E-03	MD = -0.706, p = 1.67E-13	MD = -0.948, p = 1.24E-06
OLIG2+-GAD67+	MD = -0.532, p = 0	MD = 0.046, p = 1	MD = -0.053, p = 1	MD = -0.441, p = 1.07E-08	MD = -0.741, p = 1.07E-04
ALDH1L1+-GAD67+	MD = -0.854, p = 0	MD = -0.429, p = 6.18E-02	MD = -0.491, p = 7.02E-03	MD = -0.694, p = 2.08E-13	MD = -1.015, p = 3.01E-07
IBA1+-GAD67+	MD = -0.895, p = 0	MD = -0.347, p = 1.90E-01	MD = -0.452, p = 1.40E-02	MD = -0.65, p = 7.20E-13	MD = -0.935, p = 1.63E-06
CD31+-PDGFRβ+	MD = -0.321, p = 1.27E-08	MD = -0.238, p = 5.95E-01	MD = -0.257, p = 3.28E-01	MD = -0.211, p = 3.95E-03	MD = -0.389, p = 1.04E-01
COL1A1+-PDGFRβ+	MD = -0.322, p = 1.18E-08	MD = -0.226, p = 6.48E-01	MD = -0.304, p = 1.71E-01	MD = -0.166, p = 4.03E-02	MD = -0.333, p = 2.28E-01
OLIG2+-PDGFRβ+	MD = -0.043, p = 9.61E-01	MD = 0.173, p = 8.65E-01	MD = 0.152, p = 8.50E-01	MD = 0.1, p = 4.96E-01	MD = -0.126, p = 9.68E-01
ALDH1L1+-PDGFRβ+	MD = -0.364, p = 1.75E-09	MD = -0.301, p = 3.26E-01	MD = -0.286, p = 2.23E-01	MD = -0.153, p = 7.09E-02	MD = -0.4, p = 8.73E-02
IBA1+-PDGFRβ+	MD = -0.405, p = 1.06E-10	MD = -0.219, p = 6.81E-01	MD = -0.247, p = 3.74E-01	MD = -0.11, p = 3.75E-01	MD = -0.32, p = 2.69E-01
COL1A1+-CD31+	MD = -0.001, p = 1	MD = 0.011, p = 1	MD = -0.046, p = 1	MD = 0.045, p = 9.83E-01	MD = 0.056, p = 1
OLIG2+-CD31+	MD = 0.278, p = 9.66E-07	MD = 0.411, p = 7.96E-02	MD = 0.41, p = 2.97E-02	MD = 0.31, p = 1.39E-05	MD = 0.262, p = 4.99E-01
ALDH1L1+-CD31+	MD = -0.043, p = 9.58E-01	MD = -0.063, p = 1	MD = -0.028, p = 1	MD = 0.057, p = 9.39E-01	MD = -0.011, p = 1

IBA1+-CD31+	MD = -0.084, p = 4.42E-01	MD = 0.019, p = 1	MD = 0.011, p = 1	MD = 0.101, p = 4.78E-01	MD = 0.068, p = 9.99E-01
OLIG2+-COL1A1+	MD = 0.279, p = 9.01E-07	MD = 0.4, p = 9.36E-02	MD = 0.456, p = 1.31E-02	MD = 0.265, p = 1.84E-04	MD = 0.207, p = 7.48E-01
ALDH1L1+-COL1A1+	MD = -0.042, p = 9.63E-01	MD = -0.075, p = 9.99E-01	MD = 0.018, p = 1	MD = 0.012, p = 1	MD = -0.067, p = 9.99E-01
IBA1+-COL1A1+	MD = -0.083, p = 4.56E-01	MD = 0.007, p = 1	MD = 0.057, p = 9.99E-01	MD = 0.056, p = 9.46E-01	MD = 0.013, p = 1
ALDH1L1+-OLIG2+	MD = -0.322, p = 1.04E-07	MD = -0.475, p = 3.16E-02	MD = -0.438, p = 1.81E-02	MD = -0.253, p = 3.71E-04	MD = -0.273, p = 4.50E-01
IBA1+-OLIG2+	MD = -0.362, p = 6.03E-09	MD = -0.392, p = 1.04E-01	MD = -0.399, p = 3.57E-02	MD = -0.209, p = 4.28E-03	MD = -0.194, p = 7.99E-01
IBA1+-ALDH1L1+	MD = -0.041, p = 9.76E-01	MD = 0.082, p = 9.98E-01	MD = 0.039, p = 1	MD = 0.044, p = 9.86E-01	MD = 0.079, p = 9.97E-01

Table S2 supporting Figure S5C-D: Differences in IGF-2R levels between cell types in hippocampal subregions

Differences in normalized mean IGF-2R intensity between glial and vascular cell populations across hippocampal subregions, analyzed by two-way ANOVA followed by Tukey's post hoc tests. Statistics presented for post hoc tests are mean difference (MD) between groups and p-value adjusted for multiple comparisons.

Factor	Contrast	Test statistics
Marker	Main effect	$F(5,155) = 54.753$, $p < 2E-16$
Subregion	Main effect	$F(5,155) = 4.081$, $p = 1.65E-3$
Marker * Subregion	Interaction effect	$F(25,155) = 1.731$, $p = 2.35E-2$

Marker	CD31+-PDGFRβ+	MD = -0.279, p = 4.49E-14
Marker	COL1A1+-PDGFRβ+	MD = -0.295, p = 4.55E-14
Marker	OLIG2+-PDGFRβ+	MD = -0.022, p = 9.82E-01
Marker	ALDH1L1+-PDGFRβ+	MD = -0.333, p = 2.81E-14
Marker	IBA1+-PDGFRβ+	MD = -0.374, p = 0
Marker	COL1A1+-CD31+	MD = -0.016, p = 9.95E-01
Marker	OLIG2+-CD31+	MD = 0.257, p = 7.31E-13
Marker	ALDH1L1+-CD31+	MD = -0.055, p = 4.92E-01
Marker	IBA1+-CD31+	MD = -0.095, p = 2.98E-02
Marker	OLIG2+-COL1A1+	MD = 0.273, p = 4.91E-13
Marker	ALDH1L1+-COL1A1+	MD = -0.038, p = 8.46E-01
Marker	IBA1+-COL1A1+	MD = -0.079, p = 1.56E-01
Marker	ALDH1L1+-OLIG2+	MD = -0.312, p = 4.49E-14
Marker	IBA1+-OLIG2+	MD = -0.352, p = 2.41E-14
Marker	IBA1+-ALDH1L1+	MD = -0.04, p = 8.11E-01
Subregion	CA1SR-CA1SO	MD = -0.066, p = 3.09E-01
Subregion	CA3SO-CA1SO	MD = -0.071, p = 2.12E-01
Subregion	CA3SR-CA1SO	MD = -0.088, p = 7.48E-02
Subregion	DGSM-CA1SO	MD = -0.11, p = 7.63E-03
Subregion	SLM-CA1SO	MD = -0.13, p = 7.91E-04
Subregion	CA3SO-CA1SR	MD = -0.005, p = 1
Subregion	CA3SR-CA1SR	MD = -0.022, p = 9.84E-01
Subregion	DGSM-CA1SR	MD = -0.044, p = 7.22E-01
Subregion	SLM-CA1SR	MD = -0.064, p = 3.25E-01

Subregion	CA3SR-CA3SO	MD = -0.016, p = 9.95E-01
Subregion	DGSM-CA3SO	MD = -0.039, p = 8.07E-01
Subregion	SLM-CA3SO	MD = -0.059, p = 4.06E-01
Subregion	DGSM-CA3SR	MD = -0.022, p = 9.81E-01
Subregion	SLM-CA3SR	MD = -0.042, p = 7.66E-01
Subregion	SLM-DGSM	MD = -0.02, p = 9.88E-01
Marker * Subregion	ALDH1L1+:CA1SO-CD31+:CA1SO	MD = -0.192, p = 8.25E-01
Marker * Subregion	ALDH1L1+:CA1SO-COL1A1+:CA1SO	MD = -0.052, p = 1
Marker * Subregion	ALDH1L1+:CA1SO-OLIG2+:CA1SO	MD = -0.393, p = 9.47E-04
Marker * Subregion	ALDH1L1+:CA1SO-PDGFRβ+:CA1SO	MD = -0.631, p = 2.57E-11
Marker * Subregion	CD31+:CA1SO-PDGFRβ+:CA1SO	MD = -0.439, p = 5.46E-06
Marker * Subregion	COL1A1+:CA1SO-CD31+:CA1SO	MD = -0.14, p = 9.99E-01
Marker * Subregion	COL1A1+:CA1SO-PDGFRβ+:CA1SO	MD = -0.579, p = 1.77E-08
Marker * Subregion	IBA1+:CA1SO-ALDH1L1+:CA1SO	MD = 0.026, p = 1
Marker * Subregion	IBA1+:CA1SO-CD31+:CA1SO	MD = -0.166, p = 9.61E-01
Marker * Subregion	IBA1+:CA1SO-COL1A1+:CA1SO	MD = -0.026, p = 1
Marker * Subregion	IBA1+:CA1SO-OLIG2+:CA1SO	MD = -0.368, p = 3.58E-03
Marker * Subregion	IBA1+:CA1SO-PDGFRβ+:CA1SO	MD = -0.606, p = 1.78E-10
Marker * Subregion	OLIG2+:CA1SO-CD31+:CA1SO	MD = 0.201, p = 7.44E-01
Marker * Subregion	OLIG2+:CA1SO-COL1A1+:CA1SO	MD = 0.342, p = 3.02E-02
Marker * Subregion	OLIG2+:CA1SO-PDGFRβ+:CA1SO	MD = -0.238, p = 3.72E-01
Marker * Subregion	ALDH1L1+:CA1SR-ALDH1L1+:CA1SO	MD = 0.01, p = 1
Marker * Subregion	CD31+:CA1SR-CD31+:CA1SO	MD = -0.084, p = 1
Marker * Subregion	COL1A1+:CA1SR-COL1A1+:CA1SO	MD = 0.029, p = 1

Marker * Subregion	IBA1+:CA1SR-IBA1+:CA1SO	MD = -0.054, p = 1
Marker * Subregion	OLIG2+:CA1SR-OLIG2+:CA1SO	MD = -0.051, p = 1
Marker * Subregion	PDGFRβ+:CA1SR-PDGFRβ+:CA1SO	MD = -0.196, p = 7.07E-01
Marker * Subregion	ALDH1L1+:CA1SR-CD31+:CA1SR	MD = -0.098, p = 1
Marker * Subregion	ALDH1L1+:CA1SR-COL1A1+:CA1SR	MD = -0.07, p = 1
Marker * Subregion	ALDH1L1+:CA1SR-OLIG2+:CA1SR	MD = -0.332, p = 1.92E-02
Marker * Subregion	ALDH1L1+:CA1SR-PDGFRβ+:CA1SR	MD = -0.426, p = 5.19E-05
Marker * Subregion	CD31+:CA1SR-PDGFRβ+:CA1SR	MD = -0.328, p = 5.50E-03
Marker * Subregion	COL1A1+:CA1SR-CD31+:CA1SR	MD = -0.027, p = 1
Marker * Subregion	COL1A1+:CA1SR-PDGFRβ+:CA1SR	MD = -0.355, p = 9.41E-03
Marker * Subregion	IBA1+:CA1SR-ALDH1L1+:CA1SR	MD = -0.039, p = 1
Marker * Subregion	IBA1+:CA1SR-CD31+:CA1SR	MD = -0.136, p = 9.98E-01
Marker * Subregion	IBA1+:CA1SR-COL1A1+:CA1SR	MD = -0.109, p = 1
Marker * Subregion	IBA1+:CA1SR-OLIG2+:CA1SR	MD = -0.371, p = 3.04E-03
Marker * Subregion	IBA1+:CA1SR-PDGFRβ+:CA1SR	MD = -0.464, p = 4.38E-06
Marker * Subregion	OLIG2+:CA1SR-CD31+:CA1SR	MD = 0.235, p = 4.02E-01
Marker * Subregion	OLIG2+:CA1SR-COL1A1+:CA1SR	MD = 0.262, p = 3.84E-01
Marker * Subregion	OLIG2+:CA1SR-PDGFRβ+:CA1SR	MD = -0.093, p = 1
Marker * Subregion	ALDH1L1+:CA3SO-ALDH1L1+:CA1SO	MD = 0.035, p = 1
Marker * Subregion	CD31+:CA3SO-CD31+:CA1SO	MD = -0.112, p = 1
Marker * Subregion	COL1A1+:CA3SO-COL1A1+:CA1SO	MD = -0.01, p = 1
Marker * Subregion	IBA1+:CA3SO-IBA1+:CA1SO	MD = 0.011, p = 1
Marker * Subregion	OLIG2+:CA3SO-OLIG2+:CA1SO	MD = -0.027, p = 1
Marker * Subregion	PDGFRβ+:CA3SO-PDGFRβ+:CA1SO	MD = -0.252, p = 1.72E-01

Marker * Subregion	ALDH1L1+:CA3SO-ALDH1L1+:CA1SR	MD = 0.025, p = 1
Marker * Subregion	CD31+:CA3SO-CD31+:CA1SR	MD = -0.028, p = 1
Marker * Subregion	COL1A1+:CA3SO-COL1A1+:CA1SR	MD = -0.039, p = 1
Marker * Subregion	IBA1+:CA3SO-IBA1+:CA1SR	MD = 0.065, p = 1
Marker * Subregion	OLIG2+:CA3SO-OLIG2+:CA1SR	MD = 0.023, p = 1
Marker * Subregion	PDGFRβ+:CA3SO-PDGFRβ+:CA1SR	MD = -0.056, p = 1
Marker * Subregion	ALDH1L1+:CA3SO-CD31+:CA3SO	MD = -0.045, p = 1
Marker * Subregion	ALDH1L1+:CA3SO-COL1A1+:CA3SO	MD = -0.007, p = 1
Marker * Subregion	ALDH1L1+:CA3SO-OLIG2+:CA3SO	MD = -0.331, p = 2.04E-02
Marker * Subregion	ALDH1L1+:CA3SO-PDGFRβ+:CA3SO	MD = -0.345, p = 5.26E-03
Marker * Subregion	CD31+:CA3SO-PDGFRβ+:CA3SO	MD = -0.3, p = 2.28E-02
Marker * Subregion	COL1A1+:CA3SO-CD31+:CA3SO	MD = -0.038, p = 1
Marker * Subregion	COL1A1+:CA3SO-PDGFRβ+:CA3SO	MD = -0.338, p = 3.16E-03
Marker * Subregion	IBA1+:CA3SO-ALDH1L1+:CA3SO	MD = 0.002, p = 1
Marker * Subregion	IBA1+:CA3SO-CD31+:CA3SO	MD = -0.043, p = 1
Marker * Subregion	IBA1+:CA3SO-COL1A1+:CA3SO	MD = -0.005, p = 1
Marker * Subregion	IBA1+:CA3SO-OLIG2+:CA3SO	MD = -0.329, p = 2.19E-02
Marker * Subregion	IBA1+:CA3SO-PDGFRβ+:CA3SO	MD = -0.343, p = 5.71E-03
Marker * Subregion	OLIG2+:CA3SO-CD31+:CA3SO	MD = 0.286, p = 7.62E-02
Marker * Subregion	OLIG2+:CA3SO-COL1A1+:CA3SO	MD = 0.324, p = 1.44E-02
Marker * Subregion	OLIG2+:CA3SO-PDGFRβ+:CA3SO	MD = -0.014, p = 1
Marker * Subregion	ALDH1L1+:CA3SR-ALDH1L1+:CA1SO	MD = 0.064, p = 1
Marker * Subregion	CD31+:CA3SR-CD31+:CA1SO	MD = -0.104, p = 1
Marker * Subregion	COL1A1+:CA3SR-COL1A1+:CA1SO	MD = 0.044, p = 1

Marker * Subregion	IBA1+:CA3SR-IBA1+:CA1SO	MD = -0.022, p = 1
Marker * Subregion	OLIG2+:CA3SR-OLIG2+:CA1SO	MD = -0.012, p = 1
Marker * Subregion	PDGFRβ+:CA3SR-PDGFRβ+:CA1SO	MD = -0.396, p = 9.50E-05
Marker * Subregion	ALDH1L1+:CA3SR-ALDH1L1+:CA1SR	MD = 0.054, p = 1
Marker * Subregion	CD31+:CA3SR-CD31+:CA1SR	MD = -0.02, p = 1
Marker * Subregion	COL1A1+:CA3SR-COL1A1+:CA1SR	MD = 0.015, p = 1
Marker * Subregion	IBA1+:CA3SR-IBA1+:CA1SR	MD = 0.032, p = 1
Marker * Subregion	OLIG2+:CA3SR-OLIG2+:CA1SR	MD = 0.038, p = 1
Marker * Subregion	PDGFRβ+:CA3SR-PDGFRβ+:CA1SR	MD = -0.201, p = 6.54E-01
Marker * Subregion	ALDH1L1+:CA3SR-ALDH1L1+:CA3SO	MD = 0.029, p = 1
Marker * Subregion	CD31+:CA3SR-CD31+:CA3SO	MD = 0.009, p = 1
Marker * Subregion	COL1A1+:CA3SR-COL1A1+:CA3SO	MD = 0.054, p = 1
Marker * Subregion	IBA1+:CA3SR-IBA1+:CA3SO	MD = -0.033, p = 1
Marker * Subregion	OLIG2+:CA3SR-OLIG2+:CA3SO	MD = 0.015, p = 1
Marker * Subregion	PDGFRβ+:CA3SR-PDGFRβ+:CA3SO	MD = -0.145, p = 9.89E-01
Marker * Subregion	ALDH1L1+:CA3SR-CD31+:CA3SR	MD = -0.024, p = 1
Marker * Subregion	ALDH1L1+:CA3SR-COL1A1+:CA3SR	MD = -0.032, p = 1
Marker * Subregion	ALDH1L1+:CA3SR-OLIG2+:CA3SR	MD = -0.317, p = 3.73E-02
Marker * Subregion	ALDH1L1+:CA3SR-PDGFRβ+:CA3SR	MD = -0.171, p = 9.46E-01
Marker * Subregion	CD31+:CA3SR-PDGFRβ+:CA3SR	MD = -0.147, p = 9.86E-01
Marker * Subregion	COL1A1+:CA3SR-CD31+:CA3SR	MD = 0.008, p = 1
Marker * Subregion	COL1A1+:CA3SR-PDGFRβ+:CA3SR	MD = -0.139, p = 1
Marker * Subregion	IBA1+:CA3SR-ALDH1L1+:CA3SR	MD = -0.06, p = 1
Marker * Subregion	IBA1+:CA3SR-CD31+:CA3SR	MD = -0.085, p = 1

Marker * Subregion	IBA1+:CA3SR-COL1A1+:CA3SR	MD = -0.092, p = 1
Marker * Subregion	IBA1+:CA3SR-OLIG2+:CA3SR	MD = -0.378, p = 2.18E-03
Marker * Subregion	IBA1+:CA3SR-PDGFRβ+:CA3SR	MD = -0.232, p = 4.34E-01
Marker * Subregion	OLIG2+:CA3SR-CD31+:CA3SR	MD = 0.293, p = 5.90E-02
Marker * Subregion	OLIG2+:CA3SR-COL1A1+:CA3SR	MD = 0.285, p = 3.87E-01
Marker * Subregion	OLIG2+:CA3SR-PDGFRβ+:CA3SR	MD = 0.146, p = 9.94E-01
Marker * Subregion	ALDH1L1+:DGSM-ALDH1L1+:CA1SO	MD = 0.063, p = 1
Marker * Subregion	CD31+:DGSM-CD31+:CA1SO	MD = -0.142, p = 9.92E-01
Marker * Subregion	COL1A1+:DGSM-COL1A1+:CA1SO	MD = -0.017, p = 1
Marker * Subregion	IBA1+:DGSM-IBA1+:CA1SO	MD = -0.07, p = 1
Marker * Subregion	OLIG2+:DGSM-OLIG2+:CA1SO	MD = -0.099, p = 1
Marker * Subregion	PDGFRβ+:DGSM-PDGFRβ+:CA1SO	MD = -0.325, p = 6.43E-03
Marker * Subregion	ALDH1L1+:DGSM-ALDH1L1+:CA1SR	MD = 0.053, p = 1
Marker * Subregion	CD31+:DGSM-CD31+:CA1SR	MD = -0.058, p = 1
Marker * Subregion	COL1A1+:DGSM-COL1A1+:CA1SR	MD = -0.045, p = 1
Marker * Subregion	IBA1+:DGSM-IBA1+:CA1SR	MD = -0.016, p = 1
Marker * Subregion	OLIG2+:DGSM-OLIG2+:CA1SR	MD = -0.048, p = 1
Marker * Subregion	PDGFRβ+:DGSM-PDGFRβ+:CA1SR	MD = -0.129, p = 9.98E-01
Marker * Subregion	ALDH1L1+:DGSM-ALDH1L1+:CA3SO	MD = 0.028, p = 1
Marker * Subregion	CD31+:DGSM-CD31+:CA3SO	MD = -0.03, p = 1
Marker * Subregion	COL1A1+:DGSM-COL1A1+:CA3SO	MD = -0.007, p = 1
Marker * Subregion	IBA1+:DGSM-IBA1+:CA3SO	MD = -0.081, p = 1
Marker * Subregion	OLIG2+:DGSM-OLIG2+:CA3SO	MD = -0.071, p = 1
Marker * Subregion	PDGFRβ+:DGSM-PDGFRβ+:CA3SO	MD = -0.073, p = 1

Marker * Subregion	ALDH1L1+:DGSM-ALDH1L1+:CA3SR	MD = -0.001, p = 1
Marker * Subregion	CD31+:DGSM-CD31+:CA3SR	MD = -0.038, p = 1
Marker * Subregion	COL1A1+:DGSM-COL1A1+:CA3SR	MD = -0.061, p = 1
Marker * Subregion	IBA1+:DGSM-IBA1+:CA3SR	MD = -0.048, p = 1
Marker * Subregion	OLIG2+:DGSM-OLIG2+:CA3SR	MD = -0.086, p = 1
Marker * Subregion	PDGFRβ+:DGSM-PDGFRβ+:CA3SR	MD = 0.071, p = 1
Marker * Subregion	ALDH1L1+:DGSM-CD31+:DGSM	MD = 0.013, p = 1
Marker * Subregion	ALDH1L1+:DGSM-COL1A1+:DGSM	MD = 0.028, p = 1
Marker * Subregion	ALDH1L1+:DGSM-OLIG2+:DGSM	MD = -0.232, p = 5.34E-01
Marker * Subregion	ALDH1L1+:DGSM-PDGFRβ+:DGSM	MD = -0.244, p = 3.21E-01
Marker * Subregion	CD31+:DGSM-PDGFRβ+:DGSM	MD = -0.256, p = 1.46E-01
Marker * Subregion	COL1A1+:DGSM-CD31+:DGSM	MD = -0.015, p = 1
Marker * Subregion	COL1A1+:DGSM-PDGFRβ+:DGSM	MD = -0.271, p = 8.18E-02
Marker * Subregion	IBA1+:DGSM-ALDH1L1+:DGSM	MD = -0.107, p = 1
Marker * Subregion	IBA1+:DGSM-CD31+:DGSM	MD = -0.094, p = 1
Marker * Subregion	IBA1+:DGSM-COL1A1+:DGSM	MD = -0.08, p = 1
Marker * Subregion	IBA1+:DGSM-OLIG2+:DGSM	MD = -0.339, p = 1.42E-02
Marker * Subregion	IBA1+:DGSM-PDGFRβ+:DGSM	MD = -0.351, p = 3.83E-03
Marker * Subregion	OLIG2+:DGSM-CD31+:DGSM	MD = 0.245, p = 3.11E-01
Marker * Subregion	OLIG2+:DGSM-COL1A1+:DGSM	MD = 0.26, p = 1.99E-01
Marker * Subregion	OLIG2+:DGSM-PDGFRβ+:DGSM	MD = -0.012, p = 1
Marker * Subregion	ALDH1L1+:SLM-ALDH1L1+:CA1SO	MD = 0.019, p = 1
Marker * Subregion	CD31+:SLM-CD31+:CA1SO	MD = -0.192, p = 7.48E-01
Marker * Subregion	COL1A1+:SLM-COL1A1+:CA1SO	MD = 0.075, p = 1

Marker * Subregion	IBA1+:SLM-IBA1+:CA1SO	MD = -0.07, p = 1
Marker * Subregion	OLIG2+:SLM-OLIG2+:CA1SO	MD = -0.11, p = 1
Marker * Subregion	PDGFRβ+:SLM-PDGFRβ+:CA1SO	MD = -0.429, p = 1.08E-05
Marker * Subregion	ALDH1L1+:SLM-ALDH1L1+:CA1SR	MD = 0.009, p = 1
Marker * Subregion	CD31+:SLM-CD31+:CA1SR	MD = -0.107, p = 1
Marker * Subregion	COL1A1+:SLM-COL1A1+:CA1SR	MD = 0.046, p = 1
Marker * Subregion	IBA1+:SLM-IBA1+:CA1SR	MD = -0.016, p = 1
Marker * Subregion	OLIG2+:SLM-OLIG2+:CA1SR	MD = -0.06, p = 1
Marker * Subregion	PDGFRβ+:SLM-PDGFRβ+:CA1SR	MD = -0.234, p = 3.08E-01
Marker * Subregion	ALDH1L1+:SLM-ALDH1L1+:CA3SO	MD = -0.016, p = 1
Marker * Subregion	CD31+:SLM-CD31+:CA3SO	MD = -0.079, p = 1
Marker * Subregion	COL1A1+:SLM-COL1A1+:CA3SO	MD = 0.085, p = 1
Marker * Subregion	IBA1+:SLM-IBA1+:CA3SO	MD = -0.081, p = 1
Marker * Subregion	OLIG2+:SLM-OLIG2+:CA3SO	MD = -0.083, p = 1
Marker * Subregion	PDGFRβ+:SLM-PDGFRβ+:CA3SO	MD = -0.178, p = 8.66E-01
Marker * Subregion	ALDH1L1+:SLM-ALDH1L1+:CA3SR	MD = -0.045, p = 1
Marker * Subregion	CD31+:SLM-CD31+:CA3SR	MD = -0.088, p = 1
Marker * Subregion	COL1A1+:SLM-COL1A1+:CA3SR	MD = 0.031, p = 1
Marker * Subregion	IBA1+:SLM-IBA1+:CA3SR	MD = -0.048, p = 1
Marker * Subregion	OLIG2+:SLM-OLIG2+:CA3SR	MD = -0.098, p = 1
Marker * Subregion	PDGFRβ+:SLM-PDGFRβ+:CA3SR	MD = -0.033, p = 1
Marker * Subregion	ALDH1L1+:SLM-ALDH1L1+:DGSM	MD = -0.044, p = 1
Marker * Subregion	CD31+:SLM-CD31+:DGSM	MD = -0.05, p = 1
Marker * Subregion	COL1A1+:SLM-COL1A1+:DGSM	MD = 0.091, p = 1

Marker * Subregion	IBA1+:SLM-IBA1+:DGSM	MD = 0, p = 1
Marker * Subregion	OLIG2+:SLM-OLIG2+:DGSM	MD = -0.012, p = 1
Marker * Subregion	PDGFRβ+:SLM-PDGFRβ+:DGSM	MD = -0.104, p = 1
Marker * Subregion	ALDH1L1+:SLM-CD31+:SLM	MD = 0.018, p = 1
Marker * Subregion	ALDH1L1+:SLM-COL1A1+:SLM	MD = -0.108, p = 1
Marker * Subregion	ALDH1L1+:SLM-OLIG2+:SLM	MD = -0.264, p = 2.46E-01
Marker * Subregion	ALDH1L1+:SLM-PDGFRβ+:SLM	MD = -0.183, p = 8.87E-01
Marker * Subregion	CD31+:SLM-PDGFRβ+:SLM	MD = -0.202, p = 6.45E-01
Marker * Subregion	COL1A1+:SLM-CD31+:SLM	MD = 0.126, p = 9.99E-01
Marker * Subregion	COL1A1+:SLM-PDGFRβ+:SLM	MD = -0.075, p = 1
Marker * Subregion	IBA1+:SLM-ALDH1L1+:SLM	MD = -0.063, p = 1
Marker * Subregion	IBA1+:SLM-CD31+:SLM	MD = -0.045, p = 1
Marker * Subregion	IBA1+:SLM-COL1A1+:SLM	MD = -0.171, p = 9.46E-01
Marker * Subregion	IBA1+:SLM-OLIG2+:SLM	MD = -0.327, p = 2.40E-02
Marker * Subregion	IBA1+:SLM-PDGFRβ+:SLM	MD = -0.246, p = 2.96E-01
Marker * Subregion	OLIG2+:SLM-CD31+:SLM	MD = 0.282, p = 8.83E-02
Marker * Subregion	OLIG2+:SLM-COL1A1+:SLM	MD = 0.156, p = 9.83E-01
Marker * Subregion	OLIG2+:SLM-PDGFRβ+:SLM	MD = 0.081, p = 1

Table S3 supporting Figure S5E: Differences in IGF-2R levels between glial populations in the dorsal hippocampus and corpus callosum.

Differences in raw mean IGF-2R intensity between glial populations in the dHC and CC, analyzed by two-way ANOVA followed by Tukey's post hoc tests. Statistics presented for post hoc tests are mean difference (MD) between groups and p-value adjusted for multiple comparisons.

Factor	Contrast	Statistics
Marker	Main effect	F(2,20) = 23.498, p = 5.62E-6
Region	Main effect	F(1,20) = 17.53, p = 4.54E-4
Marker * Region	Interaction effect	F(2,20) = 4.275, p = 2.85E-2
Marker	IBA1+-ALDH1L1+	MD = -0.605, p = 9.83E-01
Marker	OLIG2+-ALDH1L1+	MD = 20.319, p = 2.91E-05
Marker	OLIG2+-IBA1+	MD = 20.924, p = 1.98E-05
Region	dHC-CC	MD = 11.878, p = 4.54E-04
Marker * Region	ALDH1L1+:dHC-ALDH1L1+:CC	MD = 11.724, p = 2.08E-01
Marker * Region	IBA1+:dHC-IBA1+:CC	MD = 1.796, p = 9.99E-01
Marker * Region	OLIG2+:dHC-OLIG2+:CC	MD = 22.114, p = 2.60E-03
Marker * Region	IBA1+:dHC-ALDH1L1+:dHC	MD = -5.57, p = 8.62E-01
Marker * Region	OLIG2+:dHC-ALDH1L1+:dHC	MD = 25.513, p = 5.51E-04
Marker * Region	OLIG2+:dHC-IBA1+:dHC	MD = 31.083, p = 4.65E-05
Marker * Region	IBA1+:CC-ALDH1L1+:CC	MD = 4.359, p = 9.45E-01
Marker * Region	OLIG2+:CC-ALDH1L1+:CC	MD = 15.124, p = 5.71E-02
Marker * Region	OLIG2+:CC-IBA1+:CC	MD = 10.765, p = 2.85E-01
